# Ecomorphometric Analysis of Diversity in Cranial Shape of Pygopodid Geckos

**DOI:** 10.1093/iob/obab013

**Published:** 2021-04-22

**Authors:** George P Gurgis, Juan D Daza, Ian G Brennan, Mark Hutchinson, Aaron M Bauer, Michelle R Stocker, Jennifer C Olori

**Affiliations:** 1 Department of Biological Sciences, State University of New York at Oswego, Oswego, NY, USA; 2 Deparment of Biological Sciences, Sam Houston State University, Huntsville, TX, USA; 3 Division of Ecology & Evolution, Australian National University, Canberra, Australia; 4 Biological and Earth Sciences, South Australian Museum, Adelaide, Australia; 5 Department of Biology and Center for Biodiversity and Ecosystem Stewardship, Villanova University, Villanova, PA, USA; 6 Department of Geosciences, Virginia Tech, Blacksburg, VA, USA

## Abstract

Pygopodids are elongate, functionally limbless geckos found throughout Australia. The clade presents low taxonomic diversity (∼45 spp.), but a variety of cranial morphologies, habitat use, and locomotor abilities that vary between and within genera. In order to assess potential relationships between cranial morphology and ecology, computed tomography scans of 29 species were used for 3D geometric morphometric analysis. A combination of 24 static landmarks and 20 sliding semi-landmarks were subjected to Generalized Procrustes Alignment. Disparity in cranial shape was visualized through Principal Component Analysis, and a multivariate analysis of variance (MANOVA) was used to test for an association between shape, habitat, and diet. A subset of 27 species with well-resolved phylogenetic relationships was used to generate a phylomorphospace and conduct phylogeny-corrected MANOVA. Similar analyses were done solely on *Aprasia* taxa to explore species-level variation. Most of the variation across pygopodids was described by principal component (PC) 1(54%: cranial roof width, parabasisphenoid, and occipital length), PC2 (12%: snout elongation and braincase width), and PC3 (6%: elongation and shape of the palate and rostrum). Without phylogenetic correction, both habitat and diet were significant influencers of variation in cranial morphology. However, in the phylogeny-corrected MANOVA, habitat remained weakly significant, but not diet, which can be explained by generic-level differences in ecology rather than among species. Our results demonstrate that at higher levels, phylogeny has a strong effect on morphology, but that influence may be due to small sample size when comparing genera. However, because some closely related taxa occupy distant regions of morphospace, diverging diets, and use of fossorial habitats may contribute to variation seen in these geckos.

## Introduction

Squamates, with their high diversity (approximately 11,000 species) and extreme ecological, dietary, and locomotory variation, are a classic group used to understand morphological evolution (Uetz and Stylianou 2018). However, the size of the group can make detailed analyses of high-resolution datasets, such as computed tomography (CT), intractable, and current disputes over relationships based on molecular and morphological data (e.g., Gauthier et al. 2012; Losos et al. 2012; Wiens et al. 2012; Reeder et al. 2015) hamper our ability to investigate convergence in body form. Smaller clades within Squamata can often serve as microcosms for the larger group when they replicate similar extremes in ecological variation and morphological diversity (Webb and Shine 1994). Clades that have those characteristics are ideal for studying environmental interactions with morphological form and function because closely related taxa would share highly similar morphology if phylogenetic affinity is the only influence on shape. One such group, itself nested within the highly biodiverse gekkotans, is the Pygopodidae (flap-footed lizards). Pygopodids include 7 genera and 45 described species, with 3 monotypic genera, *Ophidiocephalus*, *Paradelma*, and *Pletholax* (Cogger 2018). Even though few species have been subject to detailed study, basic information about behavior, habitat, and diet is available for all genera, allowing preliminary study of the major adaptive trends within the clade (Jennings 2002; Wall and Shine 2013; Cogger 2018).

All pygopodids have a snake-like body plan with absent forelimbs (although pectoral girdle vestiges persist), and extremely reduced or absent hindlimbs, including a reduced pelvic girdle (Stephenson 1962; Shine 1986). Pygopodids inhabit various habitat types across Australia and parts of New Guinea, and they include an astounding array of cranial morphologies (e.g., elongate and laterally compressed, blunt, widely open, and reduced with bone loss), locomotor abilities (e.g., burrowing, ground-dwelling, and grass-swimming), prey-capture specializations, and diets (e.g., saurophagous [*Lialis*] and myrmecophagous [*Aprasia*]), reproducing much of the variation observed across squamates generally (Webb and Shine 1994). The diversification of pygopodids parallels trends present in snakes (e.g., specialization of jaw structures, jaw suspension, and tooth robustness), with pygopodids exhibiting similar behavioral and anatomical flexibility, as well as dietary specializations, through approaching snake-like body forms (Webb and Shine 1994). The pygopodid radiation also is novel among Gekkota, which despite being one of the most speciose squamate lineages, displays low ecological and morphological diversity (Webb and Shine 1994, Daza et al. 2009).

The most notable examples of specialized ecology and extreme shape variation in pygopodids are in the genera *Lialis* and *Aprasia*, which have evolved similar ecologies and morphologies to those of macrostomatan and typhlopid snakes, respectively (Patchell and Shine 1986a; Webb and Shine 1994; Daza and Bauer 2015). For example, *Lialis* preys on other lizards, usually scincids, and has evolved recurved, arched maxillae, hinged teeth, and a highly kinetic cranium with mesokinesis and streptostyly that allow ingestion of large prey (Savitzky 1981; Patchell and Shine 1986b). *Aprasia*, a primarily subterranean forager, eats various life stages of ants, and species are highly miniaturized with a reduction in dentition that is seen in other potentially convergent lineages (e.g., typhlopid, and leptotyphlopid snakes; some salamanders and extinct early tetrapods; Hanken 1984; Webb and Shine 1994; Maddin et al. 2011; Olori and Bell 2012). Based on current literature and personal observations in the field (Aaron M. Bauer, Ian G. Brennan, Mark Hutchinson, personal observations), it is unclear to what extent these geckos actively burrow. Different species of *Aprasia* were observed to burrow under the surface of the soil or perform sand-swimming behaviors, but apparently do not form their own tunnel systems, as do other head-first burrowing tetrapods such as caecilians (Ducey et al. 1993). The monotypic genus *Ophidiocephalus*, which has been observed sand-swimming, also appears to exhibit fossorial behaviors, although to a lesser extent than *Aprasia* (Mark Hutchinson, personal observation).

Although pygopodid crania were studied previously (e.g., Stephenson 1962; Kluge 1976; Greer 1989), and some species were included in broader morphometric analyses of geckos (e.g., Daza et al. 2009; Paluh and Bauer 2018), more representative studies of morphological variation solely within Pygopodidae are lacking. Our study examines differences in cranial shape across and within genera in a morphospace in order to investigate the following questions: (1) Which regions of the cranium account for most of the diversity among taxa and how has this cranial diversity accumulated through time and across lineages? (2) What are the patterns of similarity in cranial morphology observed within the morphospace? (3) Are there any ecological variables, particularly related to diet and habitat, that are correlated with patterns found in the morphospace among (a) all pygopodids and (b) at a lower taxonomic scale, within *Aprasia*, based on trends from prior studies including pygopodids (Clark et al. 2018; Laver et al. 2019).

## Materials and methods

### Specimens sampled and creation of 3D models

A total of 29 species represented by one specimen each were used; this sample covers all 7 currently recognized genera (Kluge 1976; Jennings 2002; Uetz et al. 2019). High-resolution X-ray CT scans of heads for each species were sourced from different collections such as the Western Australian Museum Database (2019; Table 1). Intraspecific variation in cranial morphology has not been studied in pygopodids, and may be a limitation to our dataset. However, at the generic level, pygopodids present large qualitative extremes in cranial structure, and we anticipate that individual variation will be much smaller than variation across this higher taxonomic level. The low sample size may more strongly affect the narrower analysis of cranial variation with *Aprasia*, but unfortunately many individual species are represented by only a small number of museum specimens. Additionally, in order to reduce the effects of ontogenetic variation, to the best of our knowledge, and as indicated by the closure of the parietal fontanelle, all specimens used in the study were adults. Avizo software (Avizo Lite, Avizo, ThermoFisher Scientific, Waltham, MA, version 9.5.0) was used to digitally isolate the bones and create a 3D model in stanford triangle file format (.ply) for each of the 29 taxa (available for download on Morphosource [www.morphosource.org]). The models were further smoothed and modified in Geomagic (Geomagic Wrap, 3D Systems, version 2017.0.1, 2017) to remove any vertebrae and the lower jaw so that only the cranium was used for morphological analysis. A resolution of around 500,000 faces was used for all models.

**Table 1:** Ecological characters and source information of pygopodid lizards

Genus	Species	Diet	Habitat	Geography	Specimen number	Data source
*Aprasia*	*aurita*	Myrmecophagous	Fossorial	East	SAMA R63331	SAMA
*Aprasia*	*clairae*	Myrmecophagous	Fossorial	West	WAM R166868	WAM
*Aprasia*	*haroldi*	Myrmecophagous	Fossorial	West	WAM R74952	WAM
*Aprasia*	*inaurita*	Myrmecophagous	Fossorial	Central	SAMA R64535	SAMA
*Aprasia*	*litorea*	Myrmecophagous	Fossorial	West	WAM R121447	WAM
*Aprasia*	*parapulchella*	Myrmecophagous	Fossorial	East	WAM R62884	WAM
*Aprasia*	*picturata*	Myrmecophagous	Fossorial	West	WAM R166877	WAM
*Aprasia*	*pseudopulchella*	Myrmecophagous	Fossorial	Central	SAMA R67733	WAM
*Aprasia*	*repens*	Myrmecophagous	Fossorial	West	CAS 104382	Bauer
*Aprasia*	*rostrata*	Myrmecophagous	Fossorial	West	WAM R142359	WAM
*Aprasia*	*smithi*	Myrmecophagous	Fossorial	West	WAM R38994	WAM
*Aprasia*	*striolata*	Myrmecophagous	Fossorial	Central	SAMA 57805	SAMA
*Delma*	*australis*	General Surface Insects	Ground	Central	SAMA 50210	SAMA
*Delma*	*borea*	General Surface Insects	Ground	Central-West	USNM 128679	USNM
*Delma*	*concinna*	General Surface Insects	Shrub	West	CUMV R-0012292	Cornell
*Delma*	*impar*	General Surface Insects	Ground	East	SAMA 55083	SAMA
*Delma*	*inornata*	General Surface Insects	Shrub	East	SAMA 62757	SAMA
*Delma*	*labialis*	General Surface Insects	Ground	East	QM 79795	QM
*Delma*	*molleri*	General Surface Insects	Ground	Central	SAMA 58266	SAMA
*Delma*	*nasuta*	General Surface Insects	Shrub	Central-West	SAMA 48820	SAMA
*Delma*	*tincta*	General Surface Insects	Ground	Central	SAMA 51553	SAMA
*Lialis*	*burtonis*	Lizards	Ground	Central/New Guinea	FMNH 166958	FMNH
*Lialis*	*jicari*	Lizards	Ground	New Guinea	SAMA 11438	SAMA
*Ophidiocephalus*	*taeniatus*	Large Insect/Arachnid Specialist	Fossorial	Central-East	SAMA R45176	SAMA
*Paradelma*	*orientalis*	General Surface Insects	Ground	East	CAS 77652	CAS
*Pletholax*	*gracilis*	General Surface Insects	Shrub	West	MCZ 187676	MCZ
*Pygopus*	*lepidopodus*	Large Insect/Arachnid Specialist	Ground	Central	CAS 135450	CAS
*Pygopus*	*nigriceps*	Large Insect/Arachnid Specialist	Ground	Central-West	CUMV R-0014267	Cornell
*Pygopus*	*schraderi*	Large Insect/Arachnid Specialist	Ground	Central-East	SAMA 65807	SAMA
*Bavaya*	*robusta*	—	—	—	CAS205423	CAS

### Landmarks

**Fig. 1:**
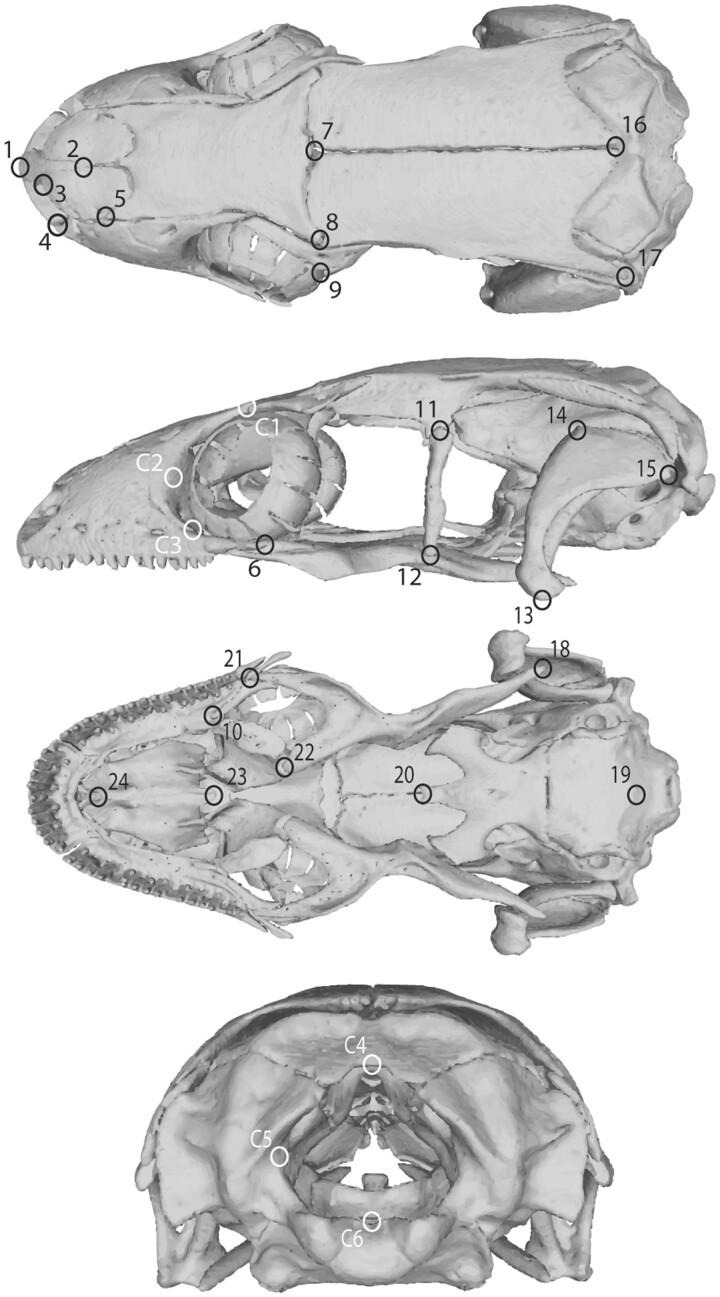
Placement of the 44 total 3D landmarks used model on the 3D model of *Paradelma orientalis* in dorsal, lateral, palatal, and posterior views. Numbers for each landmark refer to the description of the anatomical location of each landmark in Supplementary Table S1. Circles and numbers in black indicate 3D static landmarks while numbers and circles in white represent anchor points for sliding landmark curves.

Shape variation was analyzed with a 3D geometric morphometric approach using Landmark Editor version 5 software to place the landmarks (24 static landmarks and 20 sliding semi-landmarks; 44 total). Landmarks were placed on surface meshes in dorsal, left-lateral, ventral, and posterior surfaces (Fig. 1 and Supplementary Table S1). Anatomical terms used for all landmark locations were based on skull descriptions from Evans (2008). Each landmark was placed on a homologous structure solely on the left half of the cranium to avoid error due to asymmetries sometimes present in vertebrate crania (Cardini 2017; Bardua et al. 2019). Certain structures, such as the epipterygoid, were extremely reduced or absent within *Aprasia.* For the taxa in which the epipterygoid was unfeasible for landmarking, landmarks usually placed on the dorsal end of the epipterygoid were placed on the anteriormost point of the alar process of the prootic (to which the epipterygoid normally articulates), and landmarks usually placed on the ventral-most point of the epipterygoid were positioned on a retained facet in the pterygoid where the epipterygoid normally rests. Another problematic landmark was the posteriormost tip of the upper jaw. All geckos possess extremely reduced jugals that overlap the maxilla (Daza et al. 2008); for taxa in which the jugal was present, the landmark was placed on the posteriormost point of the jugal. For taxa that had a reduced, absent, or fused jugal to the ectopterygoid (Daza and Bauer 2015), the landmark was placed on the posteriormost point of the maxilla because both bones terminate at approximately the same point posteriorly in taxa that have both elements. After all landmarks were placed, landmark coordinates were mirrored along the sagittal plane of each cranium to give more representative models of the entire cranial shape. Unless the goal is to analyze differences in shape caused by asymmetry, mirroring missing landmarks has been shown as an accurate method to represent the whole cranium in shape analyses with negligible loss of information (Cardini 2017).

### Statistical analyses

Landmark coordinates were analyzed using the Geomorph statistics package (Adams et al. 2019) as well as supporting packages such as RRPP (Collyer and Adams 2018, 2019) for R software version 3.6.0 (R Core Team 2019). The code and landmark data used for the analysis have been made available on github (see Data availability statement). Landmark coordinates were subjected to Procrustes superimposition to remove the effects of size, translation, and orientation when comparing taxa so that changes in shape could be accurately observed and quantified (Zelditch et al. 2004; Klingenberg 2013). Principal component analysis (PCA) was implemented on Procrustes shape data, and the resulting morphospace was used to determine qualitative differences in morphology. Vector models (lollipop plots) using the “plotRefToTarget” function in geomorph were also generated to visualize regions where divergence from the average shape for the group was the greatest. To examine for possible allometric association of size and morphological differences, a regression was implemented using the procrustes aligned coordinates against the centroid size for each taxon.

To create a phylomorphospace we included 27 taxa sampled in the molecular phylogeny of Brennan and Oliver (2017) and constrained the topology to match the intergeneric relationships of Skipwith et al. (2019). To measure the effect of phylogenetic affinity on morphological diversity, phylogenetic signal (K) was calculated with the “physignal” function in Geomorph. As a test to compare pygopodid taxa to the typical gecko morphotype, a third morphospace was generated including a closely related gecko clade. Recent phylogenies have shown that the sister group to Pygopodidae is the Carphodactylidae (Brennan and Oliver 2017; Skipwith et al. 2019), which themselves deviate from the typical gecko cranial morphology (Paluh and Bauer 2018). Due to the specialized nature of Carphodactylidae, we elected to use the diplodactylid *Bavayia robusta*, a taxon closely related to both pygopodids and carphodactylids, which represents a more typical gecko cranial morphology (Skipwith et al. 2019).

A multivariate analysis of variance (MANOVA) was conducted in geomorph to assess whether any ecological variables were associated with the cranial diversity seen across taxa. The factors incorporated in the model are outlined below, with categories being habitat, diet, and biogeography; the null hypothesis is that no observed variation can be attributed to differences in ecology (i.e., variation within each ecological grouping). Using the same subset of 27 taxa in the phylomorphospace, a phylogeny-corrected MANOVA was also implemented to correct for the effect of phylogenetic inheritance of morphological characters.

To assess how morphological diversity has accumulated through time and is distributed among pygopodid species, we analyzed the principal component (PC) data using Disparity Through Time analyses implemented in geiger (Pennell et al. 2014) and common univariate and multivariate comparative phylogenetic models (Brownian Motion, Ornstein Uhlenbeck, and Early Burst) in MVMORPH (Clavel et al. 2015). Both of these methods provide insight into the temporal and phylogenetic partitioning of diversity early or late in clade evolution, or within or among pygopodid clades. To account for phylogenetic and divergence time uncertainty, we estimated disparity metrics and model fit on a set of 100 ultrametric trees extracted from the posterior dating analysis of Brennan and Oliver (2017).

#### Habitat categories

Pygopodids can be found across the entirety of Australia; they have been observed in almost every habitat type with some species being found in vastly different habitats across their native territories (Wall and Shine 2013). Given the wide geographic ranges and scarce accounts for the ecology of specific species, habitat groupings were based on those of Jennings (2002; Table 1) with some modifications from field observations (M. Hutchinson, personal observation). We split habitat type into the following 3 groups:

taxa with a fossorial ecology that spend the majority of their time underground. We recognize that “fossorial” is a problematic term because these species may either actively dig into loose substrate or passively use burrows built by other animals, and thus may vary in the biomechanical forces applied to their crania. However, the degree to which a species actively burrows is mostly unknown for pygopodids. Rather than make *a priori* assumptions about locomotory mode, we conservatively grouped together all endogeic species (e.g., those in soil; Bardua et al. 2019), which minimally are exposed to the same constraints imposed by tight spaces and darkness when foraging;

ground-dwelling taxa that utilize open areas on the surface, or are active on or within surface litter. Unlike Jennings (2002), we coded leaf-litter specialists as terrestrial rather than fossorial because they do not encounter the same sensory or biomechanical constraints as subterranean taxa moving through tight tunnels. However, we recognize that this distinction is somewhat artificial because many taxa may fall on a spectrum of substrate use;

species observed to travel around or within vegetation, such as spinifex grass found across Australia was coded as Shrub. This category also includes taxa described as “semi-arboreal” because they are sometimes found in the canopies of spinifex grass (e.g., *Delma concinna* and *Pletholax gracilis*; Jennings 2002).

#### Diet categories

Information about the diet of taxa was compiled from various reports in the literature (Patchell and Shine 1986a, 1986b; Webb and Shine 1994; Kutt et al. 1998; Jennings 2002; Daza et al. 2009; Wall and Shine 2013; Cogger 2018) that discussed general ecology; however, due to constraints on available information for many taxa, generalized diets were used with close consideration of extensive natural history observations (M. Hutchison, personal observation). Diet categories (Table 1) were coded into 4 groups: (1) Generalized insectivores that actively hunt insects on the surface or under leaf-litter (e.g., all *Delma*, *Pletholax*, and *Ophidiocephalus*); (2) Ambush hunters that mainly feed on other lizards (*Lialis*); (3) Large arthropod specialists that commonly feed on arachnids (e.g., many *Pygopus*); and (4) Myrmecophagous species (e.g., all *Aprasia*) observed to eat various life stages of ants (Webb and Shine 1994).

#### Biogeography

Geographic ranges for all sampled taxa were taken from Kluge (1974) and the Internation Union for Conservation of Nature (IUCN) red list database (IUCN 2019), as well as the South Australian Museum (SAMA) and Western Australian Museum (WAM) databases. A separate PCA and MANOVA were conducted with taxa the fossorial coded taxa, *Aprasia* because of the high taxonomic diversity and wide biographic distribution of this genus, as well as the genus *Ophidiocephalus*. This separate test was included as prior research suggested an unexplored qualitative association between biogeography and morphology (Clark et al. 2018; Laver et al. 2019). Geographic ranges were split into eastern (Queensland, New South Wales, Australian Capital Territory, and Victoria), western (Western Australia), and central groups (South Australia and the Northern Territory; Table 1).

#### Institutional abbreviations

Data collected from microCT scans were sourced from California Academy of Sciences, San Francisco (CAS), Museum of Comparative Zoology, Harvard University, Cambridge (MCZ), Queensland Museum (QM), SAMA, US National Museum (now National Museum of Natural History), and WAM.

## Results

### PCA and occupation in morphospace

**Fig. 2:**
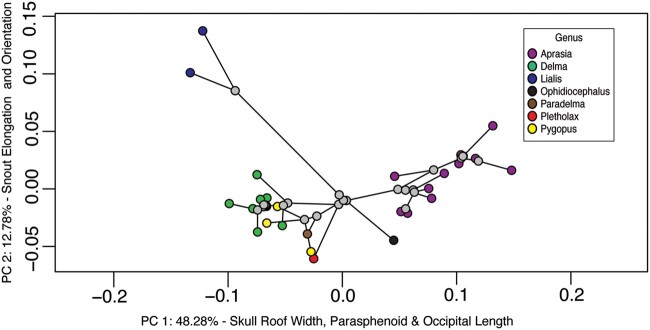
Morphospace of 27 pygopodid taxa analyzed for variation in cranial shape with known phylogenetic relationships. *B. robusta*, was included in shape analysis as an analog for more typical gecko cranial shape. Phylogenetic relationships are incorporated from molecular data of Brennan and Oliver (2017) constrained to the intergeneric topology of Skipwith (2019). Branching events are depicted as nodes indicated by white circles.

**Fig. 3:**
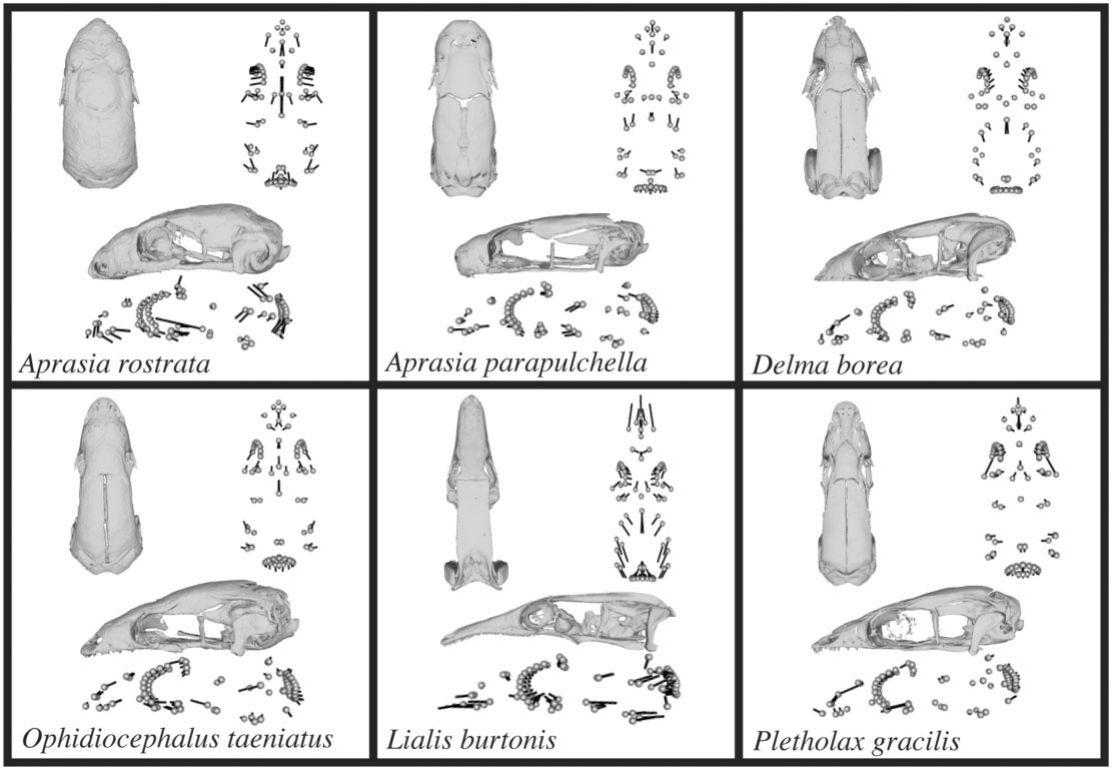
Deviation from average cranial shape of the dataset represented by vector models created in geomorph. Length and direction of lines on each model represents the magnitude and direction of deviation between the procrustes aligned coordinates and the average shape of specimens used. The figure includes one representative from *Lialis, Ophidiocephalus*, and *Pletholax. Delma borea* serves as a representative for the cluster *of Delma, Paradelma*, *and Pygopus*. Two *Aprasia* were included to show qualitative differences between geographic regions, with *A. rostrata* representing the western taxa and *A. parapulchella* representing the eastern/central taxa. Scale bars represent 5 mm in length.

Most of the variation in cranial shape (93.84%) was contained within 12 PC axes; PC1 accounted for about half (53.80%) of the variation, PC2 explained 11.93%, and PC3 explained 6.02% (remaining axes each accounted for ∼5% of variation; Supplementary Table S8). Note, that percentages are slightly different for the phylomorphospace (Fig. 2). In the morphospace, members of the same genus typically clustered together (Fig. 2). As visualized through vector models comparing individual cranium to a hypothetical average pygopodid cranium shape (Fig. 3), many *Delma* and *Paradelma* exhibit little deviation from the average of a moderately wide cranium roof, large orbits, and dorsoventrally and laterally compressed cranium compared to typical geckos (i.e., parietals longer and cranial roof not as vaulted), and moderately elongate, tapered snouts.

**Fig. 4:**
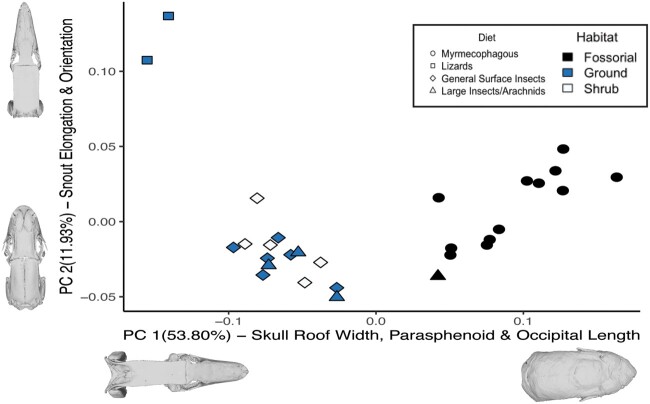
PCA of cranial shape variation in 29 pygopodid species. The numbers for each PC indicate the percentage of variance explained by each axis. Analyses for 2 descriptors, diet, and habitat, were conducted. Min and max shapes for each PC are represented as.ply files for each extreme taxon in dorsal view. Along the PC1 axis, extremes are represented by *A. rostrata* (right) and *L. burtonis* (left). Along the PC2 axis, extremes are represented by *L. jicari* (top) and *P. nigriceps* (bottom).

Among the pygopodids, *Delma concinna* (formerly *Aclys*; Kluge 1974) was isolated, although it plotted fairly close to the origin along both PC1 and PC2 and between both the other *Delma* species and the more extremely isolated *Lialis* (highly negative PC1, highly positive PC2; Figs. 2 and 4). The monotypic *Ophidiocephalus* plotted close to *Aprasia* species, suggesting similarity in cranium shape along both PC1 and PC2. There also was an unexpected, though small, separation of *Aprasia* species along PC2 that conformed with differences in biogeography and phylogeny (see below, and Table 1 for biogeographical data). When analyzed together with other pygopodid genera, all western *Aprasia* species plotted along positive PC2 space, whereas both central and eastern species were associated with more negative PC2 values. All *Aprasia* plotted in positive PC1 space, although eastern/central taxa were associated with less positive PC1 values than western species, with the exception of *A. picturata*, which shares a closer phylogenetic relationship with eastern/central species (Fig. 2 and 4).

Regarding ecology, nearly all fossorial taxa were found on the positive PC1 axis, whereas all terrestrial taxa clustered on the negative PC1 axis (Fig. 4). With respect to diet, the only clear separation was that of the myrmecophagous taxa clustering on the positive PC1 axis, while all other taxa were found along the negative PC1 axis (Fig. 4). Within the terrestrial taxa, there is a smaller separation between the lizard-specialist *Lialis* (more negative PC1 values) and the other genera. On the PC2 axis, there was no discernable pattern associated with habitat, but for diet a notable separation existed between the lizard-eaters (*Lialis*; extreme positive end of PC2) and all other pygopodids.

#### Variation captured by PCs

PC1 primarily described differences in the braincase, including the relative width of the skull roof (merged with the braincase to some degree in all pygopodids), length of the occipital region, and length of the parabasisphenoid (Fig. 4). Differences in snout elongation and width of the interorbital spacing also contribute to PC1. Along the PC1 axis, the extreme in the positive direction was *Aprasia rostrata*, which has a wider cranial table, especially in the otic region (covering the quadrates in dorsal view); more rounded braincase due to expansion of the parietal ventrolaterally and the floor of the braincase laterally and posteroventrally, becoming level with the occipital condyle in lateral view; wider interorbital spacing; and a short and blunt snout that is less dorsoventrally compressed. Additionally, the parabasisphenoid is more elongate, and the occipital region relatively shorter; overall the cranium is rounded with an “inflated” appearance. More negative PC1 values, represented by *Lialis burtonis*, correlated with a narrower cranial roof and associated braincase, especially in the otic region; narrower interorbital spacing; lengthening of the occipital region; slight narrowing of the palate; and shortening of the parabasisphenoid. Specimens at the extreme negative end of PC1 look more “box-like” and angular. The snout is highly elongated but relatively blunt because the shape differences result from lengthening along the edges as well as the tip of the snout, but the edges do not taper (i.e., are not inflected medially).

PC2 described differences mainly in the orientation and length of the snout, as well as the orbit shape, with some contributions from cranial roof shape (Fig. 4). *Pygopus nigriceps* occupied the most negative position along PC2, but much of the cranium does not differ from the average cranial shape because the majority of pygopodids were found near the origin or in positive space. *Lialis* was separated far from other pygopodids in extreme positive PC2 space, reflecting larger differences in shape. More negative PC2 values are associated with a shorter, relatively tapered snout, minor expansion and rounding of the braincase, and a larger, sub-circular orbit. At the positive extreme of PC2 was *Lialis jicari*, showing a narrower cranial roof and relatively smaller and more circular orbit, especially along the ventral rim. More positive PC2 values also are correlated with a more elongate snout-palate complex, though more tapering is exhibited because the lateral edges are elongate and deflected anteromedially, while the whole complex is angled slightly anteroventrally rather than level with the base of the orbit. There is also more dorsoventral compression of the cranium.

Variation captured by PC3 is almost entirely driven by *Pletholax*, located far from other taxa in highly negative PC3 space, whereas the other pygopodids clustered closer to the origin in positive PC3 space (Supplementary Fig. S1). Differences arise mainly from the snout; this taxon is the only pygopodid in which the ascending nasal process of the premaxilla contacts the frontal bone, separating the nasal bones. The snout is also strongly dorsoventrally compressed and the palate is elongate and narrow (Fig. 4).

### PCA of fossorial pygopodids

Variation within *Aprasia* and *Ophidiocephalus* was mostly described within the first 6 PCs (Supplementary Table S9). PC1 contributed to 27.65% of observed variation and described differences in relative length of the parabasisphenoid. PC2 contributed to 18.21% of variation and described changes of the width of the base of the snout. The extreme *A. parapulchella* on the negative end of the axis displays a less elongate braincase and a wider snout with more elongation of the parabasisphenoid and a more box-like head, compared to the extreme on the positive axis, *A. rostrata*. Although PC3 and PC4 contributed to 12 and 9% of variation, respectively, shape differences across these axes described additional, minor variations in the shape of the snout, orbit, and braincase, suggesting a generally conservative cranial shape among *Aprasia* taxa.

**Fig. 5:**
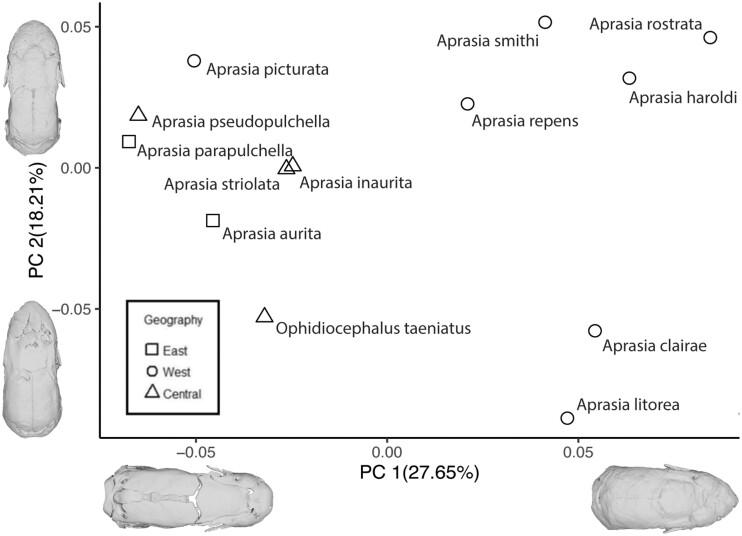
PCA of cranial shape variation within the more fossorial pygopodids. Groupings used are based on the geographic location where specimens were collected. Min and max shapes for each PC are represented as.ply files for each extreme taxon in dorsal view. Along the PC1 axis, extremes are represented by *A. rostrata* (right) and *Aprasia parapulchella* (left). Along the PC2 axis, extremes are represented by *Aprasia smithi* (top), and *Aprasia litorea* (bottom).

As in the PCA of all pygopodid taxa, there was a separation within *Aprasia* along the PC1 axis based on the geography of the native range of each species, which also falls along phylogenetic divergence within *Aprasia* (Fig. 5). Species that are found in the eastern/central parts of Australia were found to cluster separately along the negative PC1 axis, whereas species that are found in the western part of Australia cluster along the positive PC1 axis. An exception to that pattern was again *A. picturata*, which occupied positive PC1 space despite being from western Australia.

### Statistical analyses

Without phylogenetic correction, divergence in cranial morphology was explained significantly by variation in habitat (*P* < 0.005, *F* = 13.6286, df = 2) and diet (*P *< 0.005, *F* = 3.3765, df = 3) (Supplementary Table S2). Specifically within *Aprasia*, biogeography was found to explain the diversity seen across taxa found in different regions of Australia (*P* = 0.0216, *F* = 1.9426, df = 2) (Supplementary Table S4).

For the analyses that included corrections for phylogenetic relationships, habitat (*P* = 0.0119, *F* = 1.8221, df = 2) continued to hold statistical significance, but diet (*P* = 0.4156, *F* = 1.0297, df = 3), as well as biogeography for *Aprasia* (*P* = 0.2529, *F* = 1.2283, df = 2), were not found to be significant factors explaining diversity on the interspecific level (Supplementary Tables S3–S5). Phylogenetic signal was found to be a strong factor in explaining variation in cranial morphology (*P* = 0.0001, *K* = 1.43364), and this is supported by low Mean Disparity Index (MDI) values for PCs 1 (−0.25 ± 0.03) and 2 (−0.09 ± 0.04) (Supplementary Fig. S2), suggesting strong partitioning of disparity among clades. However, limited species richness and uncertainty in intergeneric relationships within the Pygopodidae likely limit confidence in our MDI estimates (PC1 *P* = 0.15 ± 0.89; PC2 *P* = 0.44 ± 0.16). Support for Early Burst models in the first 2 PCs (Supplementary Table S6) also highlights the early accumulation of diversity along these axes. In addition to the strong phylogenetic signal, the split between eastern and western taxa occurs as the main split within the tree topology for *Aprasia*.

When shape was regressed against centroid size, a clear size-related trend was revealed without (*r*^2^ = 0.41, *P* = 0.001) and weakly with (*r*^2^ = 0.09, *P* = 0.004) phylogenetic correction. Taxa with absolutely larger heads, such as *Lialis*, *Pygopus*, and *Delma* correlated with more negative PC1 values, whereas the much smaller *Aprasia* and *Ophidiocephalus* were associated with positive PC1 values.

## Discussion

### Phylogenetic patterns

Our study reveals that the patterns observed in pygopodid morphospace are most strongly influenced by phylogenetic relationships. Members of the same genus tended to cluster together (Fig. 2 and Supplemental Fig. S2), demonstrating that morphological variation at higher taxonomic levels was influenced by divergence (Sherratt et al. 2014), a pattern consistent with our low MDI estimates and support for an Early Burst model of morphological diversification. Overlap of several genera within the morphospace along both PC1 and PC2 was observed, suggesting that *Pygopus*, *Delma*, *Pletholax*, and *Paradelma* have similar cranial morphologies (Fig. 4) and may be more similar to an ancestral shape. That observation was supported by the addition of *Bavayia* into the morphospace, which while mapping separately from pygopodids as a group, was nearest the cluster comprising *Delma, Pygopus, Pletholax*, and *Paradelma* (Fig. 2).

**Fig. 6:**
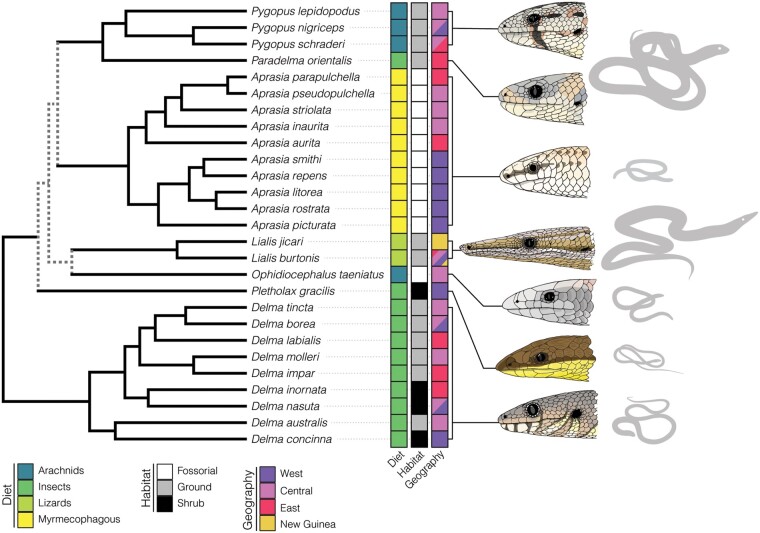
Phylogeny and ecological traits of the Pygopodidae. At the left, we show the hypothesized phylogenetic relationships of the pygopod species used in this study. Dotted gray lines indicate considerable uncertainty in the topology among genera of pygopodids. Dietary, habitat, and geographic preferences of each species are plotted to show ecological diversity. Head and body illustrations highlight the immense ecomorphological diversity of pygopodid geckos.

A strong phylogenetic signal is not surprising within a small clade because the most likely reason for closely related species to exhibit a shared morphology is inheritance from a common ancestor (i.e., phyletic inertia *sensu*Gould and Lewontin 1979). In the case of pygopodids, the ancestral limb-reduced morphology retained by all members may constrain the cranial morphology to some extent (Rieppel 1984), even within genera. On the other hand, such distinct clustering by sub-group apparently is uncommon in phylomorphospace analyses, although a similar pattern was recovered for caecilian cranial shape (Sherratt et al. 2014), another notably limb-reduced vertebrate group. Moreover, strong phylogenetic signal also was detected for PCA of head shape measurements of fossorial gymnophthalmid lizards (Barros et al. 2011), and an almost identical pattern of taxon separation to that of our morphospace was found by Kluge (1974) in a PCA of external morphological features of pygopodids. However, differing from the caecilian phylomorphospace, although members of most pygopodid genera distinctly clustered together, the majority of genera (*Delma, Paradelma, Pygopus*, and *Pletholax*) also overlapped in morphospace. Detailed examination of shape differences compared to a hypothetical average pygopodid cranium (Fig. 3) showed that these taxa are similar because they do not deviate notably from an average, ancestral form, rather than converge on a new morphotype. Overall, little convergence across taxa was detected, except among more fossorial species (i.e., those living within soil). One exception to this trend is *Delma concinna* (formerly *Aclys*), which plots separately from the other *Delma*. The original systematic placement of *D. concinna* was based on external characters, such as scalation; externally they look dissimilar to other *Delma*, but similar to other taxa such as *Pletholax*, which caused them to be placed in the monotypic genus *Aclys* (Kluge 1974). However, later morphological analyses placed *Aclys concinna* within, or at least closely related to, *Delma*, thus removing the genus *Aclys* and placing the species within *Delma* (Kluge 1976), a position reinforced by molecular data (Jennings et al. 2003; Fig. 6). The external and internal differences between *D. concinna* and other Delma species suggest a functional or ecological divergence from the rest of the genus, but unfortunately little is published on the biology of *D. concinna*.

The strong influence of phylogeny on pygopodid cranial morphology also may be explained by how ecological attributes are distributed across genera. Within each pygopodid genus, member species tend to be highly conserved ecologically (as coded in our study, given the paucity of information available for many species), and in addition, many nongeneralist habitat and diet attributes are confined to single genera. That type of bias is best exemplified by *Aprasia*, the second most speciose genus with many singular attributes that is separated from other pygopodids in the morphospace. For example, in our study, “myrmecophagy” is confined to *Aprasia*, and all included *Aprasia* species have this diet. Similarly, all 12 *Aprasia* are coded as “fossorial” for habitat, and only one taxon outside of the genus, *Ophidiocephalus taeniatus*, falls into the same category. Thus, the close alignment between genus membership and ecology in pygopodids, plus the large number of *Aprasia* species analyzed (almost half our sample), likely exacerbates the strength of the phylogenetic signal in our data.

Similar strong phylogenetic signal also was described by Daza et al. (2009) when testing the effect of diet on morphology in a geometric morphometric study of a broader sample of geckos. Evolutionarily, large changes in morphology are unlikely to occur when sister taxa occupy similar habitats and retain similar diets. Aside from *Lialis* and *Aprasia*, the 2 most widely separated genera in the morphospace and the ones with the most obvious deviations from an ancestral gecko cranial morphology (Stephenson 1962), pygopodids retain the same generalist insectivore behaviors found within most gecko lineages (Webb and Shine 1994; Daza et al. 2009), and many species share generalized habits such as locomoting in more than one way (e.g., both digging and climbing) or using multiple parts of the landscape (e.g., sand, crevices, and vegetation; Shea and Peterson 1993). The surprisingly conservative cranial shape of the majority of pygopodids also was supported by the inclusion in the PCA of the diplodactyline gecko *B. robusta*, an analog for the more “typical” gecko cranial shape (Fig. 2). *Bavayia* plotted closest to the overlapping generalist insectivores (*Pygopus*, *Delma*, *Pletholax*, and *Paradelma*), suggesting that despite their extreme elongation and limb reduction, most pygopodid genera have retained some fundamental features of gecko cranial morphology (e.g., reduction of jugal; loss of the postorbital bar and the upper temporal arch, producing a posteriorly open orbit; Stephenson 1962; Rieppel 1984; Daza et al. 2008). All of those genera also retain a relatively large orbit, as in gekkotans generally; *Pletholax* and most *Delma* are diurnal, and *Lialis* and some *Pygopus* may be active day and night. That pygopodids retain ancestral gecko features that transcend the strongly convergent influences of miniaturization, diurnality, fossoriality, and other evolutionary phenomena are further emphasized by their strongly supported position within Gekkota in phylogenetic analyses based both on molecules and on morphology, despite artificial clustering of all other limb-reduced squamate clades in morphology-based analyses (Gauthier et al. 2012).

### Allometry and miniaturization

Many pygopodids also have evolved convergent morphologies with the small, but not limb-reduced, sphaerodactylid geckos (Rieppel 1984; Daza et al. 2008, Gamble et al. 2011; Bauer et al. 2018), reflecting a history of miniaturization in the 2 groups. For example, members of both share an increased overlap of snout elements, reduction and loss of temporal elements, closure of the post-temporal fossae (i.e., closed occiput), merging of the braincase and dermal roofing bones, a broad braincase with bulging semicircular canals, and wide cranium table resulting from the relatively larger brain and sensory organs (Rieppel 1984, 1996; Daza et al. 2008; Daza and Bauer 2015; Vallejo 2018). Additionally, despite differences in cranial elongation, most of the pygopodids we studied (including the extremely elongate *Lialis*) exhibit an approximately 1:1 ratio of snout to parietal length (Fig. 3) that is also seen in sphaerodactylids (Daza et al. 2008).

Even within Pygopodidae, further size-reduction explains a large amount of observed cranial variation (Rieppel 1984). In our analysis, taxa with larger heads, such as *Lialis, Pygopus*, and *Delma*, are associated with more negative PC1 values indicative of less rounded crania with more elongate snouts (but relatively shorter parabasisphenoids), whereas the much smaller *Aprasia* and *Ophidiocephalus* plot in more positive space (Fig. 2). However, *Pletholax*, with nearly the same head size as *Ophidiocephalus*, stands out because it does not meet predictions for shape based on its size, suggesting that size alone cannot explain shape differences across Pygopodidae. The cranium of *Pletholax* is more laterally compressed and angular than expected based on sister-taxon morphology (more positive PC1 space), and it appears to be doing something different from all other pygopodids (more negative PC3 space; except possibly *Ophidiocephalus*) in the narrowness of the palate, slightly elongated parietals, and delicately constructed snout (Fig. 3 and Supplementary Fig. S1). Those differences may be associated with other unique features of *Pletholax*, including a narrower body and longer tail, and tendency to climb into the canopy of low vegetation (e.g., grass and shrubs; Greer 1989; Jennings 2002). All pygopodids seem to employ some degree of digging, but *Pletholax* may do so less often (although see descriptions of “sand-swimming”; Kluge 1974; Shea and Peterson 1993). Comparison of *Pletholax* with the other 2 smallest genera, *Aprasia* and *Ophidiocephalus*, may be informative for separating traits associated with reduced head size from those coupled with fossoriality, which often are difficult to tease apart (see Rieppel 1984, 1996; Maddin et al. 2011; Olori and Bell 2012). In our study, enlarged otic capsules (which mask the quadrate in dorsal view) are found in all 3 of the smallest pygopodid genera, whereas lengthening of the parabasisphenoid, shortening and more subterminal positioning of the snout, and broadening of the lateral braincase occur only in the more fossorial *Aprasia* and *Ophidiocephalus*).

### Ecomorphological patterns

The lack of strong deviation in diet, habitat, and cranial shape of most pygopodid genera from each other (and to some degree from that of other gekkotans) supports phylogenetic affinity being the heaviest influence on pygopodid cranial morphology. However, the few cases of convergence, in which distantly related taxa are located near each other in morphospace (e.g., *Aprasia* and *Ophidiocephalus*; *Paradelma* and *P. nigriceps*; Fig. 2), signify similarity in cranial shape and thus potential similarity in function related to ecology. As noted above, the effect of habitat was significant both with and without phylogenetic correction. Similarly, Barros et al. (2011) found that within gymnophthalmids, another group of limb-reduced, subterranean squamates with highly variable cranial morphologies (Hernández Morales, et al. 2019), diet had little effect on external head morphology, relative to microhabitat use. However, the majority of gymnophthalmids eats soft prey (similar to myrmecophagy in *Aprasia*) and show less diet variation than do pygopodid genera. Additionally, in our study, the high sample size of *Aprasia* relative to other pygopodids included in our analysis likely contributed to the strength of the relationship between cranial variation and habitat, especially when considering that taxa representing all of the nonfossorial habitat categories overlapped broadly in morphospace (Fig. 4). Although diet was not statistically significant when corrected for the strong phylogenetic signal, isolated patterns within some genera reveal that diet likely does play a limited role in pygopodid cranial variation.

One such example is the genus *Lialis*, which mapped far from other genera in morphospace (Figs. 2 and 4), an anticipated result given its extreme snout elongation and angular cranial shape (Fig. 3), but which shares a “shrub” habitat with many other pygopodids. Most other genera exhibit some degree of rounding of the cranium (most developed in *Aprasia*), likely associated with a reduction in the size of the skull, or miniaturization (e.g., Rieppel 1984, 1996; Maddin et al. 2011; Olori and Bell 2012). In terms of its habitat and substrate use, *Lialis* is generalized and shares attributes with *Pletholax* and numerous species of *Delma*. However, it departs dramatically from other pygopodids in consuming vertebrates rather than arthropods (Patchell and Shine 1986b). Natural history observations (Patchell and Shine 1986a) suggested that the long snout of *Lialis* is advantageous for capturing their favored prey, slender scincid lizards, which are held and processed via a series of lateral (transverse) jaw movements consistent with the elongation of the lateral margins of the snout identified in our study. Functionally, that snout morphology was predicted to mimic the functional advantages of macrostomatan snakes, allowing increased gape size, but achieved through a kinetic fronto-parietal joint and somewhat mobile quadrate (Patchell and Shine 1986a), rather than the greatly mobile naso-frontal joint, maxilla, and streptostylic quadrate of snakes (Gans 1961). Given the position of *Lialis* in pygopodid phylogeny (Fig. 6), we suggest that, mirroring snake evolution, the head secondarily enlarged (Fig. 3), overprinting ancestral pygopodid features related to size- and limb-reduction. Lengthening of the snout and general increase in cranial size would result in the shrinkage of the relative orbit size (also noted by Kluge 1974), as observed along PC2, which could explain why a semi-nocturnal surface-dweller possesses relatively smaller eyes than subterranean taxa (e.g., *Aprasia*). Moreover, the decreased cranial-table width observed in our data may have allowed reacquisition of mesokinesis, often lost in miniaturized taxa due to equalization of the braincase with the dermatocranium (Rieppel 1996). The close form-function relationship in *Lialis*, combined with their generalized habitat preferences, reinforces diet as the best explanation for their unique position in morphospace.

A less extreme example of the influence of diet may be apparent across the more fossorial taxa. It is clear that given their similarities along PC1 and PC2, and distant phylogenetic relationship (Fig. 2), *Aprasia* and *Ophidiocephalus* share many aspects of cranial shape due to their similar subterranean habitat. However, the 2 genera differ markedly in diet preferences. All *Aprasia* are myrmecophagous, whereas *O. taeniatus* is a large-insect and arachnid predator (Webb and Shine 1994) and plotted closer to the origin in PC1 space than did *Aprasia*. Although closest in shape to *Aprasia*, as supported by morphospace occupancy, *Ophidiocephalus* is somewhat intermediate between *Aprasia* and other pygopodids in retaining a longer, more tapered (i.e., less blunt), and terminal snout (Fig. 3), rather than the sub-terminally positioned snout of *Aprasia* and other myrmecophagous specialists (e.g., amphisbaenians, Gans 1974; typhlopid and leptotyphlopid snakes, Kley 2001; uropeltid snakes, Olori and Bell 2012). That trend across PC1 implies that *Aprasia* departs more dramatically from the basic gecko cranium at least partly because of its shift to eating only ant larvae and pupae (Webb and Shine 1994). However, that assertion is difficult to support fully because of the confounding interactions of miniaturization, ingestion of small prey, and head-first burrowing common across fossorial tetrapods (Rieppel 1984, 1996; Maddin et al. 2011; Olori and Bell 2012).

As explained above, *Aprasia, Ophidiocephalus*, and the remaining genera also form a size-series across PC1 (*Aprasia* is smallest), and additionally, all of the myrmecophagous species are *Aprasia*, bringing in phylogenetic bias (i.e., contribution of diet can be only partially tested because none of the included taxa are myrmecophagous and not *Aprasia*). Although size alone does not explain the shape variation across PC1, it could be associated with the preference for smaller prey in *Aprasia*, or perhaps more reliance on active burrowing, both of which gain an advantage from smaller size. A complicated dynamic exists across habitat, diet, and size-reduction, and our “fossorial” categorization may be too broad to capture some of the effects of habitat because it may encompass taxa along a spectrum from those that occupy existing tunnels to active burrowers. This distinction is important because taxa at the 2 extremes experience different biomechanical forces on the cranium, which also may influence cranial morphology (Gans 1974; Ducey et al. 1993). It is possible that treating locomotion or other biomechanical variables separately from habitat and diet may have better distinguished pygopodid cranial shape in our study.

### *Aprasia* and *Ophidiocephalus* biogeography

Within *Aprasia*, all taxa exhibit the same diet and are around the same size, which suggests that variation in shape associated with biogeography could be explained by biomechanical differences related to substrate use, with phylogeny taken into account. The pattern of clustering that we observed in the morphospace (Figs. 2 and 5) aligns almost exactly with *Aprasia* phylogeny (Fig. 6), in that western species and eastern/central species form 2 distinct clades. One exception is *A. picturata*, a western species that clusters with eastern/central species, especially along PC1. This suggests a biomechanical factor, because despite inherited anatomical differences in cranial structure, such as a highly reduced or absent epipterygoid, loss of the recessus scala tympani opening, and a somewhat more tapered snout in the western species, the resulting cranial shape of *A. picturata* is more similar to that of eastern/central species.

*Ophidiocephalus*, a potentially less fossorial taxon (or moving through softer substrates), similarly lacks a recessus scala tympani opening and functional epipterygoid (similar to western *Aprasia*), and yet also exhibits an unexpectedly stronger resemblance in shape to eastern/central *Aprasia*, based on morphospace position. That similarity in morphospace occupation suggests similarity in biomechanical performance, and thus *Ophidiocephalus* and eastern/central *Aprasia* may have convergently evolved a cranial shape advantageous for moving through softer substrates, but achieved through different gross anatomical modifications. Additionally, given the structural differences between most western and eastern/central *Aprasia*, it appears that higher degrees of fossoriality and some level of head-first burrowing may have evolved twice independently within the genus. The pygopodid ancestral condition of a miniaturized, limb-reduced, crevice dweller may have been taken further through differing avenues of additional size reduction and reinforcement of the cranium in the 2 *Aprasia* lineages.

### Future directions

The analysis of shape diversity across pygopodids revealed many genera occupying exclusive regions of the morphospace, indicating that this group has radiated into nonoverlapping functional units. To understand what is influencing this distinction between each genus, it is possible that other types of ecological or biological attributes (e.g., locomotion and sexual dimorphism), or synergism among them, could be better predictors for observed diversity than either habitat or diet. Future analyses that incorporate biomechanics and stress distribution in pygopodid crania could help refine explanations of the diversity between the extreme taxa and the general insectivores, especially considering that morphological differences also allow taxa to utilize less preferred prey types (Pierce et al. 2009), and that differences in habitat use may be proxies for locomotory patterns (e.g., soil compaction and burrowing). A more robust sample of crania could yield different results because multiple samples for each taxon would allow for the assessment of intraspecific variation as a source of observed diversity.

The very property that makes pygopodids interesting—a large range of morphological variation—also presents special challenges for geometric morphometrics that could introduce small errors and biases. When using landmarks it is assumed that points are placed on homologous locations (Zelditch et al. 2004). It is important to note for *Aprasia* that there were several anatomical issues that made placing landmarks difficult, so approximate locations of anatomical points were used, as described above in the Materials and Methods section. Moreover, despite focusing on only adult specimens, many pygopodids had relatively widely open sutures between the contralateral parietals as well as variable ossification of the epipterygoid (Fig. 3), which made consistent landmarking across taxa difficult, although landmarking only one side of the cranium alleviated problems with midline sutures.

## Conclusions

The main anatomical differences observed across pygopodid taxa accounting for the interspecific diversity in cranial shape include the width of the braincase, especially around the cranial roof, and anterior–posterior elongation of the snout, parabasisphenoid, and braincase. Other factors accounting for shape differences are the shape of the orbit, relative length of the occipital region, and deflection of the snout.

We found a clear distinction between fossorial and terrestrial taxa. However, that diversity may be a result of functional demands on the cranium because some, but not all, fossorial pygopodids may dig with their heads. Within *Aprasia*, there was a correlation between biogeography, phylogeny, and cranial morphology. Our results described a divergence between Eastern/Central and Western taxa, with Eastern/Central species sharing an overall shape similarity with *O. taeniatus*. Subsequently, even though diet was not found to be statistically significant once phylogenetic correction was added, general insectivores, myrmecophages, and saurophages all clustered separately within the morphospace. However, diet primarily was grouped by genus, explaining why phylogeny was found to be the biggest descriptor of variation in morphology.

The nature of pygopodids as a whole being ecologically and morphologically divergent from other geckos, as well as highly disparate across genera, provides a clade that can serve as a microcosm for understanding the influence of environmental interaction on morphological traits. More detailed information on ecology, behavior, and cranial function of pygopodids is needed to elucidate the influences on cranial diversity observed within this clade. The more extreme morphologies seen within pygopodids, such as *Lialis* and *Aprasia*, can help us understand this phenomenon through comparison with sister groups exhibiting less derived anatomical traits. Once more is known about the secretive lifestyles of the Pygopodidae, these geckos can serve as a model for morphological radiations in other geckos, and possibly different squamates lineages.

## Supplementary Material

obab013_Supplementary_Data
