# Supplementary material for: Ecomorphometric Analysis of Diversity in Cranial Shape of Pygopodid Geckos
Source: Integr Org Biol. 2021 Apr 22;3(1):obab013. doi: 10.1093/iob/obab013 (PMC8341893; doi:10.1093/iob/obab013)
Supplement: obab013_Supplementary_Data [file obab013_supplementary_data.zip › Figure S2.docx]

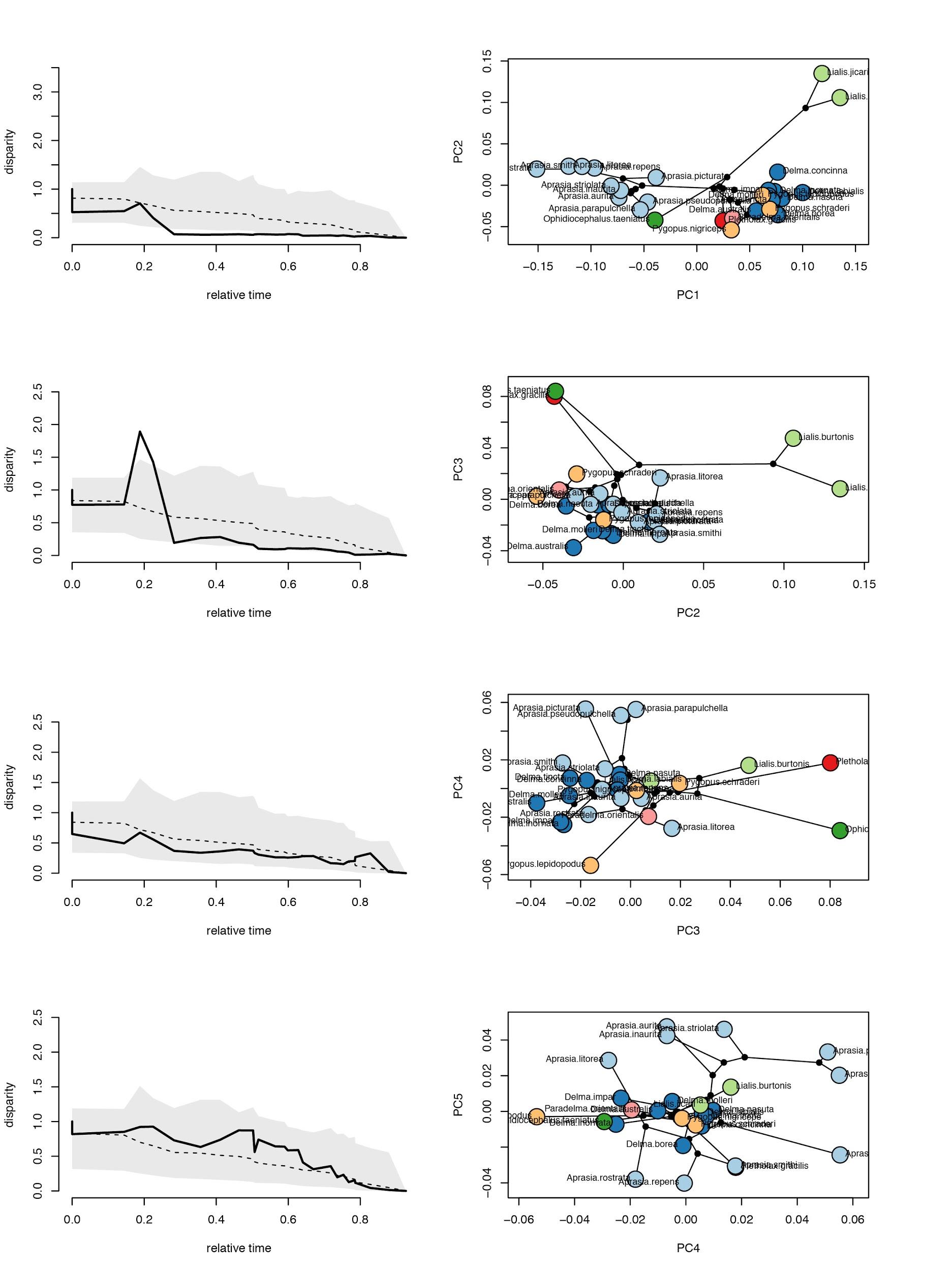


**Figure S2**. Temporal and phylogenetic visualizations of the first four Principal Component axes. On the left, disparity through time plots generated using the R package ‘geiger’ show the first two PC axes have low mean disparity indexes suggesting disparity in these traits are concentrated among clades (primarily genera), whereas PC axes three and four follow the null model of Brownian Motion evolution more closely. On the right, pairwise plots of the first four PC axes highlight the separate clustering of genera, except for generalist species in *Delma* and *Pygopus*.
