# Supplementary material for: Ecomorphometric Analysis of Diversity in Cranial Shape of Pygopodid Geckos
Source: Integr Org Biol. 2021 Apr 22;3(1):obab013. doi: 10.1093/iob/obab013 (PMC8341893; doi:10.1093/iob/obab013)
Supplement: obab013_Supplementary_Data [file obab013_supplementary_data.zip › Supplementary Data.docx]

# **Supplementary Data**

**Table S1.** Description of Landmark Locations. Landmarks indicated with S are static landmarks while those indicated with C are anchor points for sliding semi-landmark curves. Landmark locations on .ply model example seen in fig 1 of the text.

| **Landmark** | **View** | **Description** |
| --- | --- | --- |
| S1 | Dorsal | Anterior-most tip of the premaxilla |
| S2 | Dorsal | Posterior-most tip of the nasal process of the premaxilla |
| S3 | Dorsal | Triple point of contact among the nasal, premaxilla, and external nares |
| S4 | Dorsal | Lateral-most contact of the premaxilla and maxilla |
| S5 | Dorsal | Triple point of contact between the nasal, maxilla, and frontal |
| S6 | Lateral | Posterior-most point of the upper jaw |
| S7 | Dorsal | Midpoint of the frontoparietal suture |
| S8 | Dorsal | Triple point of contact between the frontal, parietal, and postorbitofrontal |
| S9 | Dorsal | Lateral-most point of the postorbitofrontal |
| S10 | Ventral | Anterior-most point of the ectopterygoid |
| S11 | Lateral | Dorsal-most tip of the epipterygoid |
| S12 | Lateral | Ventral-most tip of the epipterygoid |
| S13 | Lateral | Ventral-most point of the mandibular condyle of the quadrate |
| S14 | Lateral | Anterodorsal-most point of the tympanic crest of the quadrate |
| S15 | Lateral | Posterior-most point of the cephalic condyle of the quadrate |
| S16 | Dorsal | Medial extent of the posterior edge of the parietal |
| S17 | Dorsal | Posterior-most point of the postparietal process of the parietal |
| S18 | Ventral | Posterior-most point of the quadrate process of the pterygoid |
| S19 | Ventral | Midline of the parabasisphenoid |
| S20 | Ventral | Anterior-most tip of the parabasisphenoid of the braincase |
| S21 | Ventral | Anterior-most tip of the ectopterygoid process of the pterygoid |
| S22 | Ventral | Anterior-most tip of the palatine process of the pterygoid |
| S23 | Ventral | Posterior-most point of the palatine process of the vomer |
| S24 | Ventral | Anterior-most point of the suture between the contralateral vomers |
| C1 | Lateral | Posterior-most tip of the dorsal process of the prefrontal |
| C2 | Lateral | Midpoint of the posterior edge of the prefrontal |
| C3 | Lateral | Posterior-most connection of the prefrontal and and maxilla |
| C4 | Posterior | Midpoint of the dorsal rim of the foramen magnum |
| C5 | Posterior | Midpoint of the lateral rim of the foramen magnum |
| C6 | Posterior | Posteromedial-most point of the occipital condyle |

**Table S2.** MANOVA results for influence of diet and habitat on morphological traits without phylogenetic correction

|  | DF | SS | MS | Rsq | F | Z | Pr(>F) |
| --- | --- | --- | --- | --- | --- | --- | --- |
| Diet | 3 | 0.06816 | 0.022720 | 0.17279 | 3.874 | 4.1996 | 0.05 > |
| Habitat | 2 | 0.19142 | 0.095708 | 0.48525 | 16.319 | 5.7321 | 0.05 > |
| Diet*Habitat | 1 | 0.00634 | 0.006345 | 0.01494 | 0.8411 | -0.1836 | 0.5647 |
| Residuals | 23 | 0.13489 | 0.005865 | 0.34196 |  |  |  |
| Total | 28 | 0.39447 |  |  |  |  |  |

**Table S3.** MANOVA results for influence of diet and habitat on morphological traits with phylogenetic correction

|  | DF | SS | MS | Rsq | F | Z | Pr(>F) |
| --- | --- | --- | --- | --- | --- | --- | --- |
| Diet | 3 | 0.5937 | 0.19789 | 0.11138 | 1.0297 | 0.19922 | 0.4156 |
| Habitat | 2 | 0.7004 | 0.35018 | 0.13140 | 1.8221 | 2.25426 | 0.0119 |
| Diet*Habitat | 1 | 1.2120 | 0.24240 | 0.2274 | 1.2362 | 1.0934 | 0.137 |
| Residuals | 21 | 4.0359 | 0.19219 | 0.75722 | - | - | - |
| Total | 26 | 5.3300 | - | - | - | - | - |

**Table S4.** MANOVA results for influence of biogeography on morphological traits in the fossorial pygopodids without phylogenetic correction.

|  | DF | SS | MS | Rsq | F | Z | Pr(>F) |
| --- | --- | --- | --- | --- | --- | --- | --- |
| Geography | 3 | 0.045656 | 0.0152186 | 0.36903 | 1.7546 | 2.3513 | 0.0113 |
| Residuals | 9 | 0.078063 | 0.0086737 | 0.63097 | - | - | - |
| Total | 12 | 0.123719 | - | - | - | - | - |

**Table S5.** MANOVA results for influence of biogeography on morphological traits in the fossorial pygopodids with phylogenetic correction

|  | DF | SS | MS | Rsq | F | Z | Pr(>F) |
| --- | --- | --- | --- | --- | --- | --- | --- |
| Geography | 3 | 0.6677 | 0.22257 | 0.19983 | 0.666 | -1.2478 | 0.8852 |
| Residuals | 8 | 2.6737 | 0.33421 | 0.80017 | - | - | - |
| Total | 11 | 3.3414 | - | - | - | - | - |

**Table S6.** Phylogenetic comparative model fitting results of the first ten PC axes.

| PCs | Model | AIC | ΔAIC |
| --- | --- | --- | --- |
| PC1 | BM | -92.90413 | 2.7004471 |
| PC1 | OU | -90.90171 | 4.7028632 |
| **PC1** | **EB** | **-95.60457** | **0** |
| PC2 | BM | -107.8292 | 5.3054262 |
| PC2 | OU | -105.8218 | 7.3129152 |
| **PC2** | **EB** | **-113.1347** | **0** |
| PC3 | BM | -105.1878 | 2.2885891 |
| **PC3** | **OU** | **-107.4763** | **0** |
| PC3 | EB | -103.1881 | 4.2882357 |
| PC4 | BM | -100.9448 | 4.6641419 |
| **PC4** | **OU** | **-115.609** | **0** |
| PC4 | EB | -98.94485 | 6.6641201 |
| PC1-2 | BM | -201.991 | 8.379754 |
| PC1-2 | OU | -195.9911 | 4.37967 |
| **PC1-2** | **EB** | **-210.3707** | **0** |
| PC1-3 | BM | -303.5822 | 0.2882423 |
| PC1-3 | OU | -296.4319 | 7.4385417 |
| **PC1-3** | **EB** | **-303.8704** | **0** |
| PC1-4 | BM | -408.3339 | 1.4118667 |
| **PC1-4** | **OU** | **-409.7457** | **0** |
| PC1-4 | EB | -407.9182 | 1.8275369 |
| **PC1-10** | **BM** | **-1122.641** | **0** |
| PC1-10 | OU | -1113.22 | 9.4203726 |
| PC1-10 | EB | -1119.927 | 2.7135766 |
|  |  |  |  |

**Table S7.** Morphosource links for all specimens used in the study.

**Table S8.** Summary of PCA for the morphospace of all taxa. Only principal components that contributed to more than 5% of variance were included in interpretation of shape variation.

|  | PC1 | PC2 | PC3 | PC4 | PC5 | PC6 | PC7 | PC8 | PC9 | PC10 | PC 11 | PC 12 |
| --- | --- | --- | --- | --- | --- | --- | --- | --- | --- | --- | --- | --- |
| Standard Deviation | 0.09043 | 0.04259 | 0.03026 | 0.02650 | 0.02466 | 0.02271 | 0.01957 | 0.01849 | 0.01584 | 0.01493 | 0.01358 | 0.01224 |
| Proportion of Variance | 0.53796 | 0.11931 | 0.06023 | 0.04618 | 0.04000 | 0.03392 | 0.02520 | 0.02249 | 0.01650 | 0.01466 | 0.01213 | 0.00985 |
| Cumulative Proportion | 0.53796 | 0.65727 | 0.71750 | 0.76368 | 0.80367 | 0.83759 | 0.86279 | 0.88528 | 0.90178 | 0.91644 | 0.92857 | 0.93842 |

**Table S9.** Summary of PCA for the fossorial pygopodid morphospace. Only principal components that contributed to more than 5% of variance were included in interpretation of shape variation.

|  | PC1 | PC2 | PC3 | PC4 | PC5 | PC6 |
| --- | --- | --- | --- | --- | --- | --- |
| Standard Deviation | 0.05339 | 0.04333 | 0.03671 | 0.03442 | 0.02889 | 0.025886 |
| Proportion of Variance | 0.27651 | 0.18213 | 0.13072 | 0.11490 | 0.08095 | 0.06500 |
| Cumulative Proportion | 0.27651 | 0.45864 | 0.58935 | 0.70426 | 0.78521 | 0.85021 |

| Species | Source | Morphosource Link |
| --- | --- | --- |
| *Aprasia aurita* | sama:herpetology:r63331 | <https://www.morphosource.org/Detail/MediaDetail/Show/media_id/78861> |
| *Aprasia clairae* | WAM:R:166868 | <https://www.morphosource.org/Detail/MediaDetail/Show/media_id/78862> |
| *Aprasia haroldi* | wam:rept:r103982 | <https://www.morphosource.org/Detail/MediaDetail/Show/media_id/78865> |
| *Aprasia inaurita* | SAMA:R:54535 | <https://www.morphosource.org/Detail/MediaDetail/Show/media_id/78868> |
| *Aprasia litorea* | wam:rept:r121447 | <https://www.morphosource.org/Detail/MediaDetail/Show/media_id/78872> |
| *Aprasia parapulchella* | wam:rept:r62884 | <https://www.morphosource.org/Detail/MediaDetail/Show/media_id/78873> |
| *Aprasia picturata* | wam:rept:r166877 | <https://www.morphosource.org/Detail/MediaDetail/Show/media_id/78874> |
| *Aprasia pseudopulchella* | sama:herpetology:r67733 | <https://www.morphosource.org/Detail/MediaDetail/Show/media_id/78875> |
| *Aprasia repens* | cas:herp:104382 | <https://www.morphosource.org/Detail/MediaDetail/Show/media_id/78876> |
| *Aprasia rostrata* | wam:rept:r153828 | <https://www.morphosource.org/Detail/MediaDetail/Show/media_id/78878> |
| *Aprasia smithi* | wam:rept:r38994 | <https://www.morphosource.org/Detail/MediaDetail/Show/media_id/78880> |
| *Aprasia striolata* | sama:herpetology:r57805 | <https://www.morphosource.org/Detail/MediaDetail/Show/media_id/78879> |
| *Bavayia robusta* | cas:herp:205423 | <https://www.morphosource.org/Detail/MediaDetail/Show/media_id/78871> |
| *Delma australis* | SAMA:R:50210 | <https://www.morphosource.org/Detail/MediaDetail/Show/media_id/78847> |
| *Delma borea* | usnm:amphibians & reptiles:128679 | <https://www.morphosource.org/Detail/MediaDetail/Show/media_id/78850> |
| *Delma concinna* | cumv:herps:r-0012292 | <https://www.morphosource.org/Detail/MediaDetail/Show/media_id/78851> |
| *Delma impar* | SAMA:R:55083 | <https://www.morphosource.org/Detail/MediaDetail/Show/media_id/78848> |
| *Delma inornata* | SAMA:R:62757 | <https://www.morphosource.org/Detail/MediaDetail/Show/media_id/78849> |
| *Delma labialis* | QM:J:79795 | <https://www.morphosource.org/Detail/MediaDetail/Show/media_id/78845> |
| *Delma molleri* | SAMA:R:58266 | <https://www.morphosource.org/Detail/MediaDetail/Show/media_id/78852> |
| *Delma nasuta* | SAMA:R:48820 | <https://www.morphosource.org/Detail/MediaDetail/Show/media_id/78853> |
| *Delma tincta* | SAMA:R:51553 | <https://www.morphosource.org/Detail/MediaDetail/Show/media_id/78854> |
| *Lialis burtonis* | fmnh:amphibians and reptiles:166958 | <https://www.morphosource.org/Detail/MediaDetail/Show/media_id/78855> |
| *Lialis jicari* | SAMA:R:11438 | <https://www.morphosource.org/Detail/MediaDetail/Show/media_id/78856> |
| *Ophidiocephalus taeniatus* | SAMA:R:45179 | <https://www.morphosource.org/Detail/MediaDetail/Show/media_id/78846> |
| *Paradelma orientalis* | cas:herp:77652 | <https://www.morphosource.org/Detail/MediaDetail/Show/media_id/78859> |
| *Pletholax gracilis* | mcz:herp:r-187676 | <https://www.morphosource.org/Detail/MediaDetail/Show/media_id/78844> |
| *Pygopus lepidopodus* | cas:herp:135450 | <https://www.morphosource.org/Detail/MediaDetail/Show/media_id/78860> |
| *Pygopus nigriceps* | CUMV:R:0014267 | <https://www.morphosource.org/Detail/MediaDetail/Show/media_id/78858> |
| *Pygopus schraderi* | SAMA:R:65807 | <https://www.morphosource.org/Detail/MediaDetail/Show/media_id/78857> |


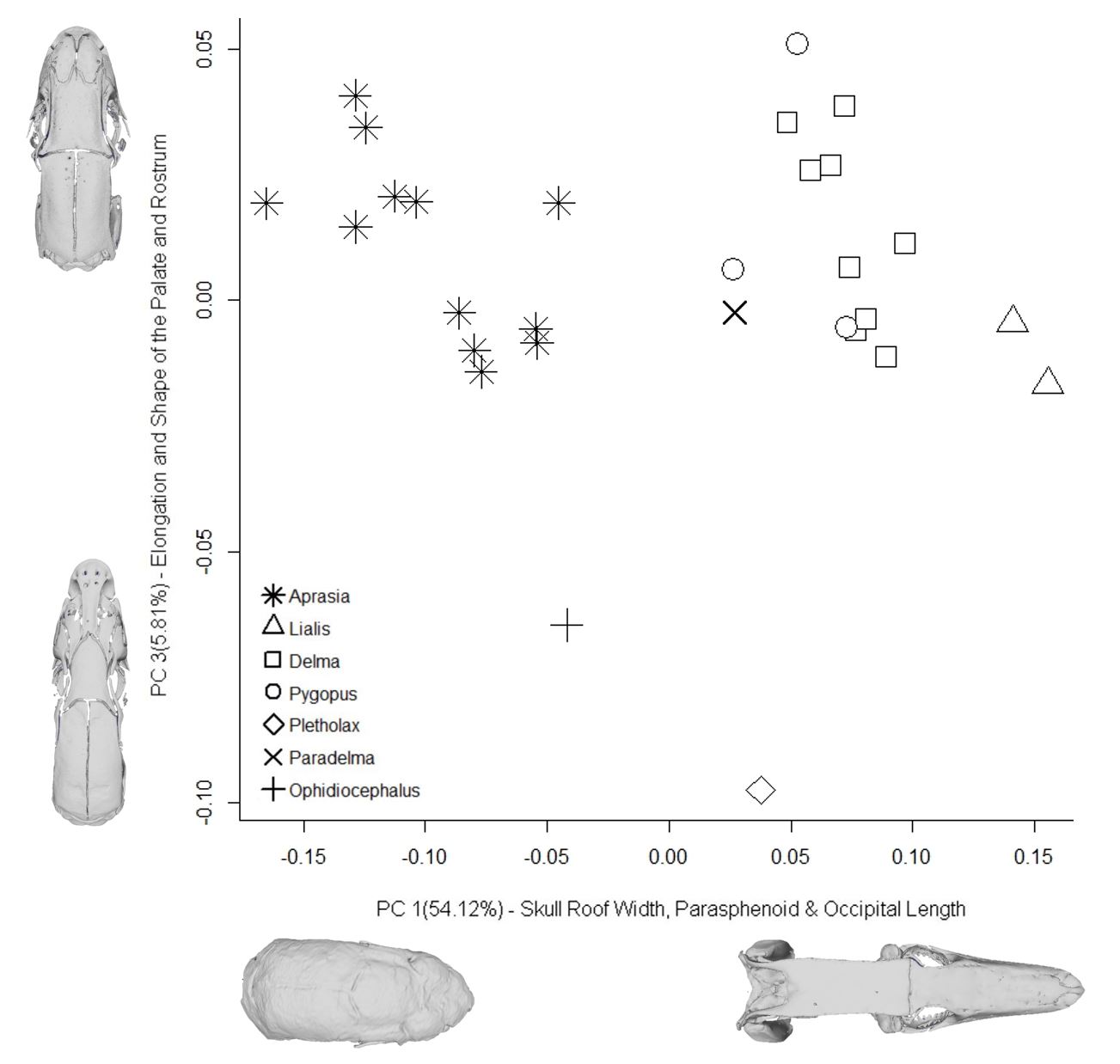
**Figure S1**. Morphospace of all taxa comparing PC1 vs. PC3


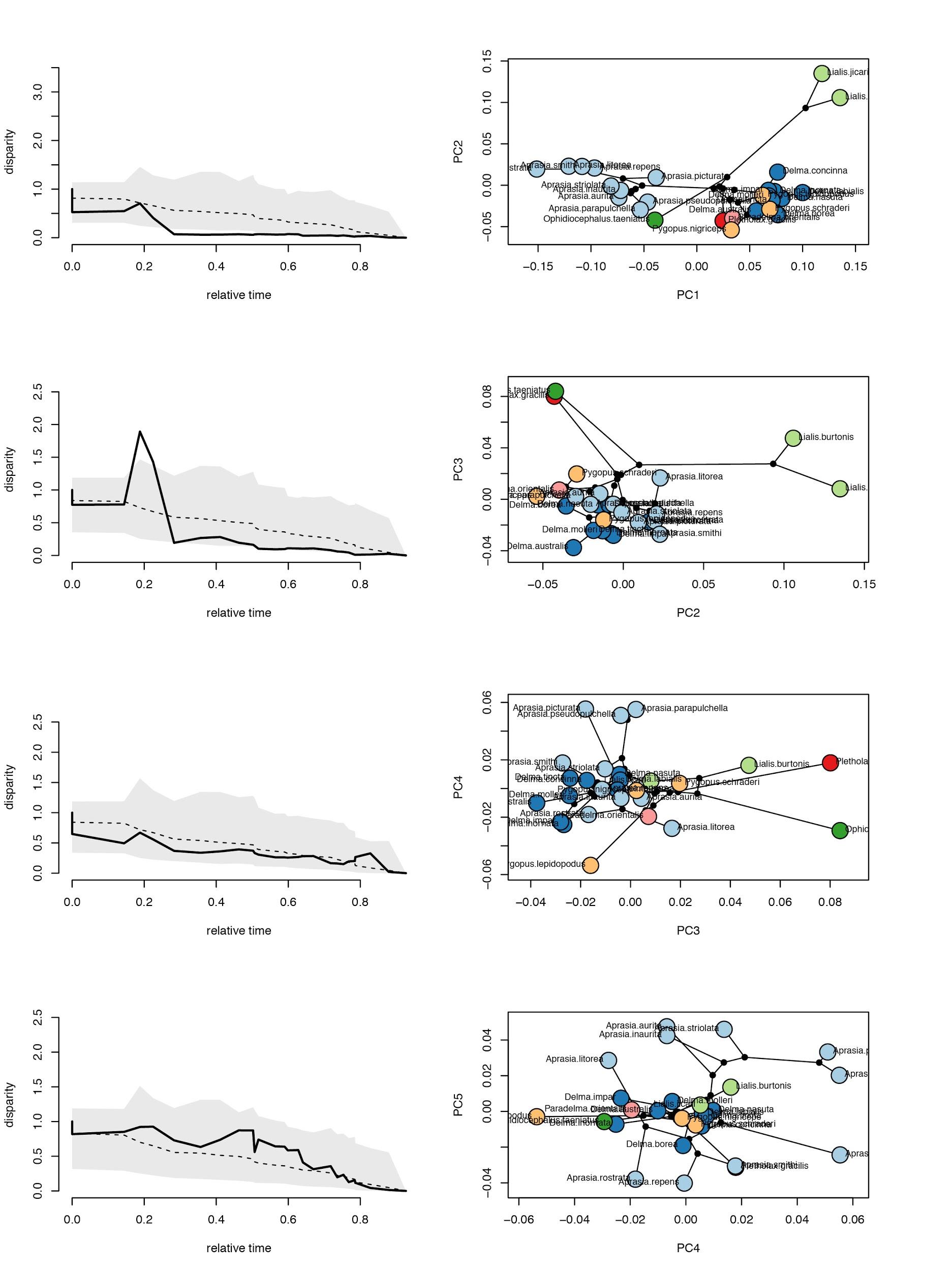


**Figure S2**. Temporal and phylogenetic visualizations of the first four Principal Component axes. On the left, disparity through time plots generated using the R package ‘geiger’ show the first two PC axes have low mean disparity indexes suggesting disparity in these traits are concentrated among clades (primarily genera), whereas PC axes three and four follow the null model of Brownian Motion evolution more closely. On the right, pairwise plots of the first four PC axes highlight the separate clustering of genera, except for generalist species in *Delma* and *Pygopus*.
