# Supplementary material for: Ecomorphometric Analysis of Diversity in Cranial Shape of Pygopodid Geckos
Source: Integr Org Biol. 2021 Apr 22;3(1):obab013. doi: 10.1093/iob/obab013 (PMC8341893; doi:10.1093/iob/obab013)
Supplement: obab013_Supplementary_Data [file obab013_supplementary_data.zip › Table S1.docx]

**Table S1.** Description of Landmark Locations. Landmarks indicated with S are static landmarks while those indicated with C are anchor points for sliding semi-landmark curves. Landmark locations on .ply model example seen in fig 1 of the text.

| **Landmark** | **View** | **Description** |
| --- | --- | --- |
| S1 | Dorsal | Anterior-most tip of the premaxilla |
| S2 | Dorsal | Posterior-most tip of the nasal process of the premaxilla |
| S3 | Dorsal | Triple point of contact among the nasal, premaxilla, and external nares |
| S4 | Dorsal | Lateral-most contact of the premaxilla and maxilla |
| S5 | Dorsal | Triple point of contact between the nasal, maxilla, and frontal |
| S6 | Lateral | Posterior-most point of the upper jaw |
| S7 | Dorsal | Midpoint of the frontoparietal suture |
| S8 | Dorsal | Triple point of contact between the frontal, parietal, and postorbitofrontal |
| S9 | Dorsal | Lateral-most point of the postorbitofrontal |
| S10 | Ventral | Anterior-most point of the ectopterygoid |
| S11 | Lateral | Dorsal-most tip of the epipterygoid |
| S12 | Lateral | Ventral-most tip of the epipterygoid |
| S13 | Lateral | Ventral-most point of the mandibular condyle of the quadrate |
| S14 | Lateral | Anterodorsal-most point of the tympanic crest of the quadrate |
| S15 | Lateral | Posterior-most point of the cephalic condyle of the quadrate |
| S16 | Dorsal | Medial extent of the posterior edge of the parietal |
| S17 | Dorsal | Posterior-most point of the postparietal process of the parietal |
| S18 | Ventral | Posterior-most point of the quadrate process of the pterygoid |
| S19 | Ventral | Midline of the parabasisphenoid |
| S20 | Ventral | Anterior-most tip of the parabasisphenoid of the braincase |
| S21 | Ventral | Anterior-most tip of the ectopterygoid process of the pterygoid |
| S22 | Ventral | Anterior-most tip of the palatine process of the pterygoid |
| S23 | Ventral | Posterior-most point of the palatine process of the vomer |
| S24 | Ventral | Anterior-most point of the suture between the contralateral vomers |
| C1 | Lateral | Posterior-most tip of the dorsal process of the prefrontal |
| C2 | Lateral | Midpoint of the posterior edge of the prefrontal |
| C3 | Lateral | Posterior-most connection of the prefrontal and and maxilla |
| C4 | Posterior | Midpoint of the dorsal rim of the foramen magnum |
| C5 | Posterior | Midpoint of the lateral rim of the foramen magnum |
| C6 | Posterior | Posteromedial-most point of the occipital condyle |
