# Supplementary material for: Ecomorphometric Analysis of Diversity in Cranial Shape of Pygopodid Geckos
Source: Integr Org Biol. 2021 Apr 22;3(1):obab013. doi: 10.1093/iob/obab013 (PMC8341893; doi:10.1093/iob/obab013)
Supplement: obab013_Supplementary_Data [file obab013_supplementary_data.zip › Table S2.docx]

**Table S2.** MANOVA results for influence of diet and habitat on morphological traits without phylogenetic correction

|  | DF | SS | MS | Rsq | F | Z | Pr(>F) |
| --- | --- | --- | --- | --- | --- | --- | --- |
| Diet | 3 | 0.06816 | 0.022720 | 0.17279 | 3.874 | 4.1996 | 0.05 > |
| Habitat | 2 | 0.19142 | 0.095708 | 0.48525 | 16.319 | 5.7321 | 0.05 > |
| Diet*Habitat | 1 | 0.00634 | 0.006345 | 0.01494 | 0.8411 | -0.1836 | 0.5647 |
| Residuals | 23 | 0.13489 | 0.005865 | 0.34196 |  |  |  |
| Total | 28 | 0.39447 |  |  |  |  |  |
