# Supplementary material for: Ecomorphometric Analysis of Diversity in Cranial Shape of Pygopodid Geckos
Source: Integr Org Biol. 2021 Apr 22;3(1):obab013. doi: 10.1093/iob/obab013 (PMC8341893; doi:10.1093/iob/obab013)
Supplement: obab013_Supplementary_Data [file obab013_supplementary_data.zip › Table S3.docx]

**Table S3.** MANOVA results for influence of diet and habitat on morphological traits with phylogenetic correction

|  | DF | SS | MS | Rsq | F | Z | Pr(>F) |
| --- | --- | --- | --- | --- | --- | --- | --- |
| Diet | 3 | 0.5937 | 0.19789 | 0.11138 | 1.0297 | 0.19922 | 0.4156 |
| Habitat | 2 | 0.7004 | 0.35018 | 0.13140 | 1.8221 | 2.25426 | 0.0119 |
| Diet*Habitat | 1 | 1.2120 | 0.24240 | 0.2274 | 1.2362 | 1.0934 | 0.137 |
| Residuals | 21 | 4.0359 | 0.19219 | 0.75722 | - | - | - |
| Total | 26 | 5.3300 | - | - | - | - | - |
