# Supplementary material for: Ecomorphometric Analysis of Diversity in Cranial Shape of Pygopodid Geckos
Source: Integr Org Biol. 2021 Apr 22;3(1):obab013. doi: 10.1093/iob/obab013 (PMC8341893; doi:10.1093/iob/obab013)
Supplement: obab013_Supplementary_Data [file obab013_supplementary_data.zip › Table S4.docx]

**Table S4.** MANOVA results for influence of biogeography on morphological traits in the fossorial pygopodids without phylogenetic correction.

|  | DF | SS | MS | Rsq | F | Z | Pr(>F) |
| --- | --- | --- | --- | --- | --- | --- | --- |
| Geography | 3 | 0.045656 | 0.0152186 | 0.36903 | 1.7546 | 2.3513 | 0.0113 |
| Residuals | 9 | 0.078063 | 0.0086737 | 0.63097 | - | - | - |
| Total | 12 | 0.123719 | - | - | - | - | - |
