# Supplementary material for: Ecomorphometric Analysis of Diversity in Cranial Shape of Pygopodid Geckos
Source: Integr Org Biol. 2021 Apr 22;3(1):obab013. doi: 10.1093/iob/obab013 (PMC8341893; doi:10.1093/iob/obab013)
Supplement: obab013_Supplementary_Data [file obab013_supplementary_data.zip › Table S5.docx]

**Table S5.** MANOVA results for influence of biogeography on morphological traits in the fossorial pygopodids with phylogenetic correction

|  | DF | SS | MS | Rsq | F | Z | Pr(>F) |
| --- | --- | --- | --- | --- | --- | --- | --- |
| Geography | 3 | 0.6677 | 0.22257 | 0.19983 | 0.666 | -1.2478 | 0.8852 |
| Residuals | 8 | 2.6737 | 0.33421 | 0.80017 | - | - | - |
| Total | 11 | 3.3414 | - | - | - | - | - |
