# Supplementary material for: Ecomorphometric Analysis of Diversity in Cranial Shape of Pygopodid Geckos
Source: Integr Org Biol. 2021 Apr 22;3(1):obab013. doi: 10.1093/iob/obab013 (PMC8341893; doi:10.1093/iob/obab013)
Supplement: obab013_Supplementary_Data [file obab013_supplementary_data.zip › Table S6.docx]

**Table S6.** Phylogenetic comparative model fitting results of the first ten PC axes.

| PCs | Model | AIC | ΔAIC |
| --- | --- | --- | --- |
| PC1 | BM | -92.90413 | 2.7004471 |
| PC1 | OU | -90.90171 | 4.7028632 |
| **PC1** | **EB** | **-95.60457** | **0** |
| PC2 | BM | -107.8292 | 5.3054262 |
| PC2 | OU | -105.8218 | 7.3129152 |
| **PC2** | **EB** | **-113.1347** | **0** |
| PC3 | BM | -105.1878 | 2.2885891 |
| **PC3** | **OU** | **-107.4763** | **0** |
| PC3 | EB | -103.1881 | 4.2882357 |
| PC4 | BM | -100.9448 | 4.6641419 |
| **PC4** | **OU** | **-115.609** | **0** |
| PC4 | EB | -98.94485 | 6.6641201 |
| PC1-2 | BM | -201.991 | 8.379754 |
| PC1-2 | OU | -195.9911 | 4.37967 |
| **PC1-2** | **EB** | **-210.3707** | **0** |
| PC1-3 | BM | -303.5822 | 0.2882423 |
| PC1-3 | OU | -296.4319 | 7.4385417 |
| **PC1-3** | **EB** | **-303.8704** | **0** |
| PC1-4 | BM | -408.3339 | 1.4118667 |
| **PC1-4** | **OU** | **-409.7457** | **0** |
| PC1-4 | EB | -407.9182 | 1.8275369 |
| **PC1-10** | **BM** | **-1122.641** | **0** |
| PC1-10 | OU | -1113.22 | 9.4203726 |
| PC1-10 | EB | -1119.927 | 2.7135766 |
|  |  |  |  |
