# Supplementary material for: Ecomorphometric Analysis of Diversity in Cranial Shape of Pygopodid Geckos
Source: Integr Org Biol. 2021 Apr 22;3(1):obab013. doi: 10.1093/iob/obab013 (PMC8341893; doi:10.1093/iob/obab013)
Supplement: obab013_Supplementary_Data [file obab013_supplementary_data.zip › Table S7.docx]

**Table S7.** Morphosource links for all specimens used in the study.

| Species | Source | Morphosource Link |
| --- | --- | --- |
| *Aprasia aurita* | sama:herpetology:r63331 | <https://www.morphosource.org/Detail/MediaDetail/Show/media_id/78861> |
| *Aprasia clairae* | WAM:R:166868 | <https://www.morphosource.org/Detail/MediaDetail/Show/media_id/78862> |
| *Aprasia haroldi* | wam:rept:r103982 | <https://www.morphosource.org/Detail/MediaDetail/Show/media_id/78865> |
| *Aprasia inaurita* | SAMA:R:54535 | <https://www.morphosource.org/Detail/MediaDetail/Show/media_id/78868> |
| *Aprasia litorea* | wam:rept:r121447 | <https://www.morphosource.org/Detail/MediaDetail/Show/media_id/78872> |
| *Aprasia parapulchella* | wam:rept:r62884 | <https://www.morphosource.org/Detail/MediaDetail/Show/media_id/78873> |
| *Aprasia picturata* | wam:rept:r166877 | <https://www.morphosource.org/Detail/MediaDetail/Show/media_id/78874> |
| *Aprasia pseudopulchella* | sama:herpetology:r67733 | <https://www.morphosource.org/Detail/MediaDetail/Show/media_id/78875> |
| *Aprasia repens* | cas:herp:104382 | <https://www.morphosource.org/Detail/MediaDetail/Show/media_id/78876> |
| *Aprasia rostrata* | wam:rept:r153828 | <https://www.morphosource.org/Detail/MediaDetail/Show/media_id/78878> |
| *Aprasia smithi* | wam:rept:r38994 | <https://www.morphosource.org/Detail/MediaDetail/Show/media_id/78880> |
| *Aprasia striolata* | sama:herpetology:r57805 | <https://www.morphosource.org/Detail/MediaDetail/Show/media_id/78879> |
| *Bavayia robusta* | cas:herp:205423 | <https://www.morphosource.org/Detail/MediaDetail/Show/media_id/78871> |
| *Delma australis* | SAMA:R:50210 | <https://www.morphosource.org/Detail/MediaDetail/Show/media_id/78847> |
| *Delma borea* | usnm:amphibians & reptiles:128679 | <https://www.morphosource.org/Detail/MediaDetail/Show/media_id/78850> |
| *Delma concinna* | cumv:herps:r-0012292 | <https://www.morphosource.org/Detail/MediaDetail/Show/media_id/78851> |
| *Delma impar* | SAMA:R:55083 | <https://www.morphosource.org/Detail/MediaDetail/Show/media_id/78848> |
| *Delma inornata* | SAMA:R:62757 | <https://www.morphosource.org/Detail/MediaDetail/Show/media_id/78849> |
| *Delma labialis* | QM:J:79795 | <https://www.morphosource.org/Detail/MediaDetail/Show/media_id/78845> |
| *Delma molleri* | SAMA:R:58266 | <https://www.morphosource.org/Detail/MediaDetail/Show/media_id/78852> |
| *Delma nasuta* | SAMA:R:48820 | <https://www.morphosource.org/Detail/MediaDetail/Show/media_id/78853> |
| *Delma tincta* | SAMA:R:51553 | <https://www.morphosource.org/Detail/MediaDetail/Show/media_id/78854> |
| *Lialis burtonis* | fmnh:amphibians and reptiles:166958 | <https://www.morphosource.org/Detail/MediaDetail/Show/media_id/78855> |
| *Lialis jicari* | SAMA:R:11438 | <https://www.morphosource.org/Detail/MediaDetail/Show/media_id/78856> |
| *Ophidiocephalus taeniatus* | SAMA:R:45179 | <https://www.morphosource.org/Detail/MediaDetail/Show/media_id/78846> |
| *Paradelma orientalis* | cas:herp:77652 | <https://www.morphosource.org/Detail/MediaDetail/Show/media_id/78859> |
| *Pletholax gracilis* | mcz:herp:r-187676 | <https://www.morphosource.org/Detail/MediaDetail/Show/media_id/78844> |
| *Pygopus lepidopodus* | cas:herp:135450 | <https://www.morphosource.org/Detail/MediaDetail/Show/media_id/78860> |
| *Pygopus nigriceps* | CUMV:R:0014267 | <https://www.morphosource.org/Detail/MediaDetail/Show/media_id/78858> |
| *Pygopus schraderi* | SAMA:R:65807 | <https://www.morphosource.org/Detail/MediaDetail/Show/media_id/78857> |
