# Supplementary material for: Ecomorphometric Analysis of Diversity in Cranial Shape of Pygopodid Geckos
Source: Integr Org Biol. 2021 Apr 22;3(1):obab013. doi: 10.1093/iob/obab013 (PMC8341893; doi:10.1093/iob/obab013)
Supplement: obab013_Supplementary_Data [file obab013_supplementary_data.zip › Table S8.docx]

**Table S8.** Summary of PCA for the morphospace of all taxa. Only principal components that contributed to more than 5% of variance were included in interpretation of shape variation.

|  | PC1 | PC2 | PC3 | PC4 | PC5 | PC6 | PC7 | PC8 | PC9 | PC10 | PC 11 | PC 12 |
| --- | --- | --- | --- | --- | --- | --- | --- | --- | --- | --- | --- | --- |
| Standard Deviation | 0.09043 | 0.04259 | 0.03026 | 0.02650 | 0.02466 | 0.02271 | 0.01957 | 0.01849 | 0.01584 | 0.01493 | 0.01358 | 0.01224 |
| Proportion of Variance | 0.53796 | 0.11931 | 0.06023 | 0.04618 | 0.04000 | 0.03392 | 0.02520 | 0.02249 | 0.01650 | 0.01466 | 0.01213 | 0.00985 |
| Cumulative Proportion | 0.53796 | 0.65727 | 0.71750 | 0.76368 | 0.80367 | 0.83759 | 0.86279 | 0.88528 | 0.90178 | 0.91644 | 0.92857 | 0.93842 |
