# Supplementary material for: Ecomorphometric Analysis of Diversity in Cranial Shape of Pygopodid Geckos
Source: Integr Org Biol. 2021 Apr 22;3(1):obab013. doi: 10.1093/iob/obab013 (PMC8341893; doi:10.1093/iob/obab013)
Supplement: obab013_Supplementary_Data [file obab013_supplementary_data.zip › Table S9.docx]

**Table S9.** Summary of PCA for the fossorial pygopodid morphospace. Only principal components that contributed to more than 5% of variance were included in interpretation of shape variation.

|  | PC1 | PC2 | PC3 | PC4 | PC5 | PC6 |
| --- | --- | --- | --- | --- | --- | --- |
| Standard Deviation | 0.05339 | 0.04333 | 0.03671 | 0.03442 | 0.02889 | 0.025886 |
| Proportion of Variance | 0.27651 | 0.18213 | 0.13072 | 0.11490 | 0.08095 | 0.06500 |
| Cumulative Proportion | 0.27651 | 0.45864 | 0.58935 | 0.70426 | 0.78521 | 0.85021 |
