# Supplementary figures and images for: Ecomorphometric Analysis of Diversity in Cranial Shape of Pygopodid Geckos
Source: Integr Org Biol. 2021 Apr 22;3(1):obab013. doi: 10.1093/iob/obab013 (PMC8341893; doi:10.1093/iob/obab013)

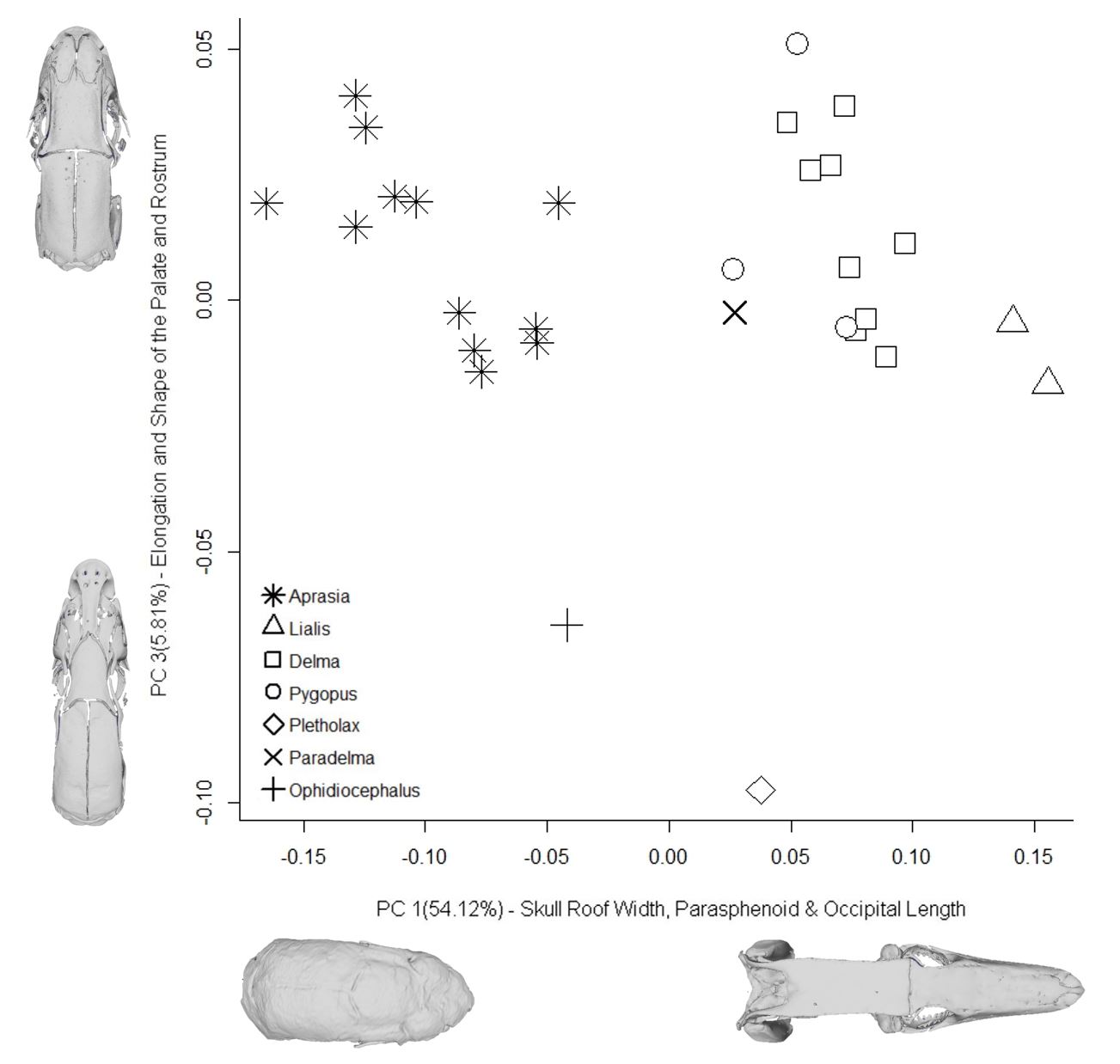
**Figure S1**. Morphospace of all taxa comparing PC1 vs. PC3

Supplement: obab013_Supplementary_Data [file obab013_supplementary_data.zip › Figure S1.docx]
